# Supplementary material for: Exploitation of the Cooperative Behaviors of Anti-CRISPR Phages
Source: Cell Host Microbe. 2020 Feb 12;27(2):189–198.e6. doi: 10.1016/j.chom.2019.12.004 (PMC7013381; doi:10.1016/j.chom.2019.12.004)
Supplement: Document S1. Figures S1–S6 and Tables S1 and S2 [file mmc1.pdf]

Cell Host & Microbe, Volume *27*

Supplemental Information

Exploitation of the Cooperative Behaviors of Anti-CRISPR Phages

Anne Chevallereau, Sean Meaden, Olivier Fradet, Mariann Landsberger, Alice Maestri, Ambarish Biswas, Sylvain Gandon, Stineke van Houte, and Edze R. Westra

Supplementary Figures

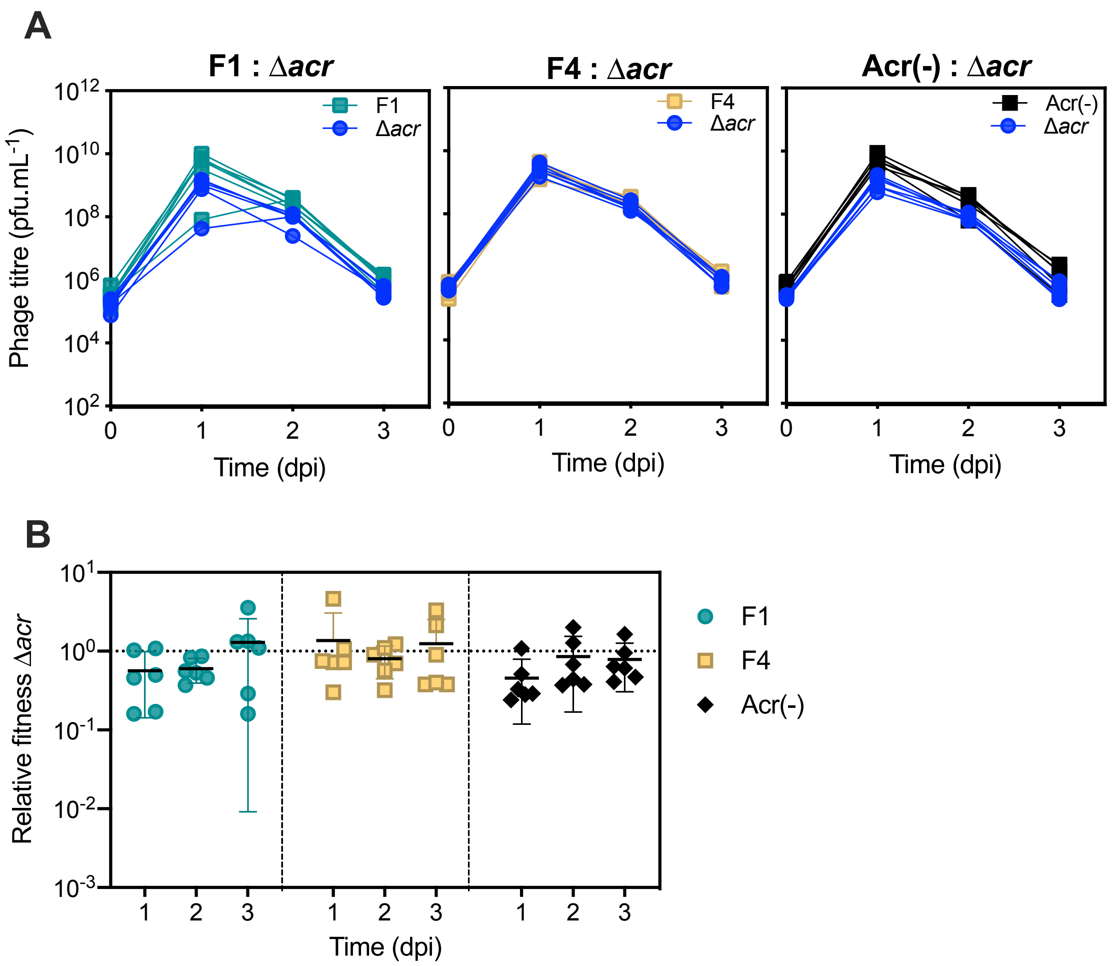

Figure S1. Carrying *acr* operon is not associated with fitness costs. Related to Figure 2. Phage mutants lacking the *acr* operon (Δ*acr*) were co-cultured for 3 days with either Acr-positive phages encoding the strong AcrIF1 (F1) or the weak AcrIF4 (F4) or with Acr-negative phages encoding AcrIE3 (Acr(-)), inactive against the CRISPR-Cas system of WT PA14. The host population carries a non-functional CRISPR-Cas system (CRISPR-KO). (**A**) Titres of each phage type were determined by spot assay on indicator strains. **(B)** Fitness values of Δ*acr*-phage relative to that of indicated phage competitors were calculated each day (with fitness at T=0 set as a reference). Panels show individual data (A) and mean (B) from 6 biological replicates. Errors bars show 95% c.i.

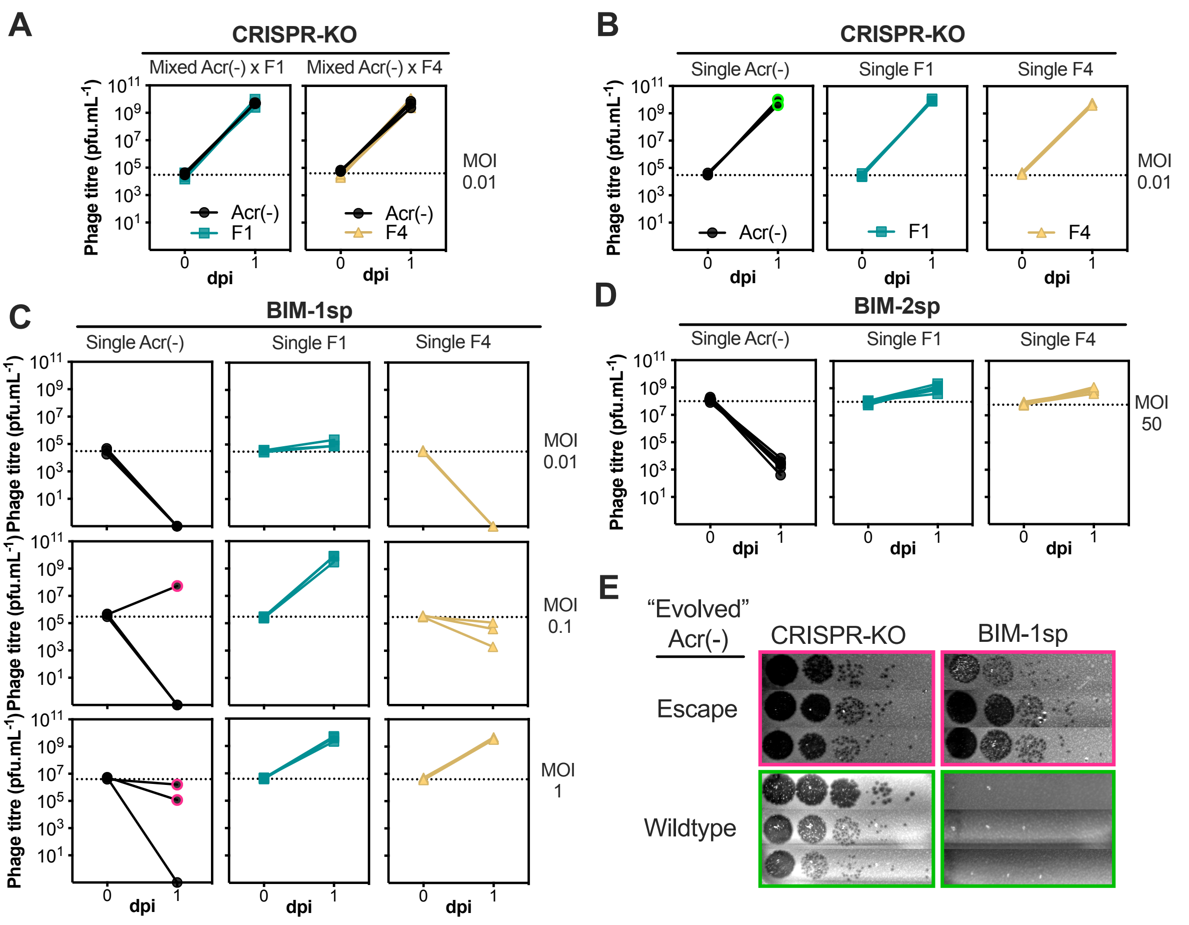

Figure S2. Control phage amplification during individual or mixed infection of CRISPR-KO or BIM host. Related to Figure 4.

Amplification of Acr-negative and Acr-positive phages during (**A**) mixed or (**B**) individual infection of CRISPR-KO host, (**C**) individual infection of CRISPR-resistant host BIM-1sp or (**D**) BIM 2sp. Initial MOI are indicated. (**E**) Acr-negative phages that amplified/maintained after 24h individual infection of CRISPR-KO (green circled dots) or BIM-1sp (pink circled dots) were isolated and the ability of these 'evolved' Acr-negative phages to infect BIM-1sp on their own was assessed (EOP assay). Individual data from 3 biological replicates are shown.

A

Acr(-):F1 – BIM-1sp (MOI 1)

Protospacer 1

ancestral Acr(-)	CC**AACGGCCGACGCTTCTGGGTCGTCGTGAAAGT**
1dpi-Acr(-)_A1	CCAACGGCCGACGCTTCTGGGTCGTCGTGAAAGT
1dpi-Acr(-)_A2	CCAACGGCCGACGCTTCTGGGTCGTCGTGAAAGT
1dpi-Acr(-)_A3	CCAACGGCCGACGCTTCTGGGTCGTCGTGAAAGT
1dpi-Acr(-)_A4	CCAACGGCCGACGCTTCTGGGTCGTCGTGAAAGT
1dpi-Acr(-)_A5	CCAACGGCCGACGCTTCTGGGTCGTCGTGAAAGT
1dpi-Acr(-)_A6	CCAACGGCCGACGCTTCTGGGTCGTCGTGAAAGT

B

Acr(-):F4 – BIM-1sp (MOI 0.1 – MOI 1)

Protospacer 1

ancestral Acr(-)	CC**AACGGCCGACGCTTCTGGGTCGTCGTGAAAGT**
1dpi-Acr(-)_C1	CCA**G**CGGCCGACGCTTCTGGGTCGTCGTGAAAGT
1dpi-Acr(-)_C2	C**G**AACGGCCGACGCTTCTGGGTCGTCGTGAAAGT
1dpi-Acr(-)_C3	CCA**G**CGGCCGACGCTTCTGGGTCGTCGTGAAAGT
1dpi-Acr(-)_C4	C**T**AACGGCCGACGCTTCTGGGTCGTCGTGAAAGT
1dpi-Acr(-)_C5	CCAACG**C**CCGACGCTTCTGGGTCGTCGTGAAAGT
1dpi-Acr(-)_C6	C**T**AACGGCCGACGCTTCTGGGTCGTCGTGAAAGT

C

Acr(-):F1 – BIM-2sp (MOI 1)

Protospacer 1

ancestral Acr(-)	CC**AACGGCCGACGCTTCTGGGTCGTCGTGAAAGT**
1dpi-Acr(-)_B1	CCAACGGCCGACGCTTCTGGGTCGTCGTGAAAGT
1dpi-Acr(-)_B2	CCAACGGCCGACGCTTCTGGGTCGTCGTGAAAGT
1dpi-Acr(-)_B3	CCAACGGCCGACGCTTCTGGGTCGTCGTGAAAGT
1dpi-Acr(-)_B4	CCAACGGCCGACGCTTCTGGGTCGTCGTGAAAGT
1dpi-Acr(-)_B5	CCAACGGCCGACGCTTCTGGGTCGTCGTGAAAGT

Protospacer 2

ancestral Acr(-)	CC**TGGACAACGGCAGCGCGATTCCCCGCGTGACC**
1dpi-Acr(-)_B1	CCTGGACAACGGCAGCGCGATTCCCCGCGTGACC
1dpi-Acr(-)_B2	CCTGGACAACGGCAGCGCGATTCCCCGCGTGACC
1dpi-Acr(-)_B3	CCTGGACAACGGCAGCGCGATTCCCCGCGTGACC
1dpi-Acr(-)_B4	CCTGGACAACGGCAGCGCGATTCCCCGCGTGACC
1dpi-Acr(-)_B5	CCTGGACAACGGCAGCGCGATTCCCCGCGTGACC

Figure S3. Protospacer sequences of Acr-negative phages. Related to Figure 4.

Acr-negative phages that amplified on CRISPR-resistant host carrying (**A,B**) 1 spacer (BIM-1sp) or (**C**) 2 spacers (BIM-2sp) were recovered at 1 dpi and phage protospacers were sequenced. Panels (**A**) and (**C**) show Acr-negative phages grown in presence of strong Acr-phage F1 (isolated from experiments of Fig. 4a and b, respectively (green circled dots)). Panel (**B**) shows Acr-negative phages cultured in presence of weak Acr-phage F4 (isolated from experiment presented in Fig. 4c (pink circled dots)). Protospacers of wildtype DMS3*vir* are indicated in bold letters and protospacer adjacent motifs are underlined. Point escape mutations are highlighted in red in panel b. Sequences shown correspond to phages isolated from 5 or 6 biological replicate experiments.

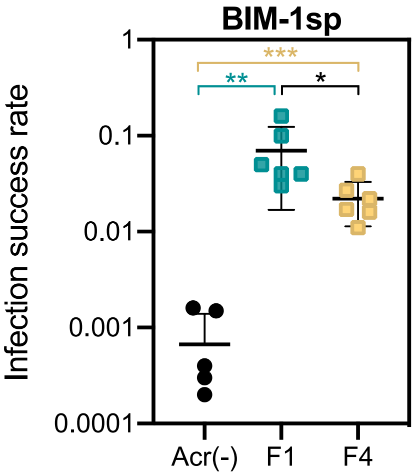

Figure S4. Experimental estimation of parameter ϕ. Related to Figure 5.

An estimation of parameter ϕ was achieved by measuring the ECOI of phages DMS3*vir*, DMS3*vir-acrIF1* and DMS3*vir-acrIF4* (indicated as Acr(-), F1 and F4, respectively) on CRISPR-resistant host BIM-1sp (one targeting spacer) and CRISPR-KO host. Asterisks indicate significant differences (two tailed t-tests): Acr(-)/F1: p=0.007, t_{10}=3.36; Acr(-)/F4: p=0.0005, t_{10}=5.10; F1/F4: p=0.05, t_{10}=2.27. Individual values and means from 6 biological replicates are shown. Error bars represent 95% c.i.

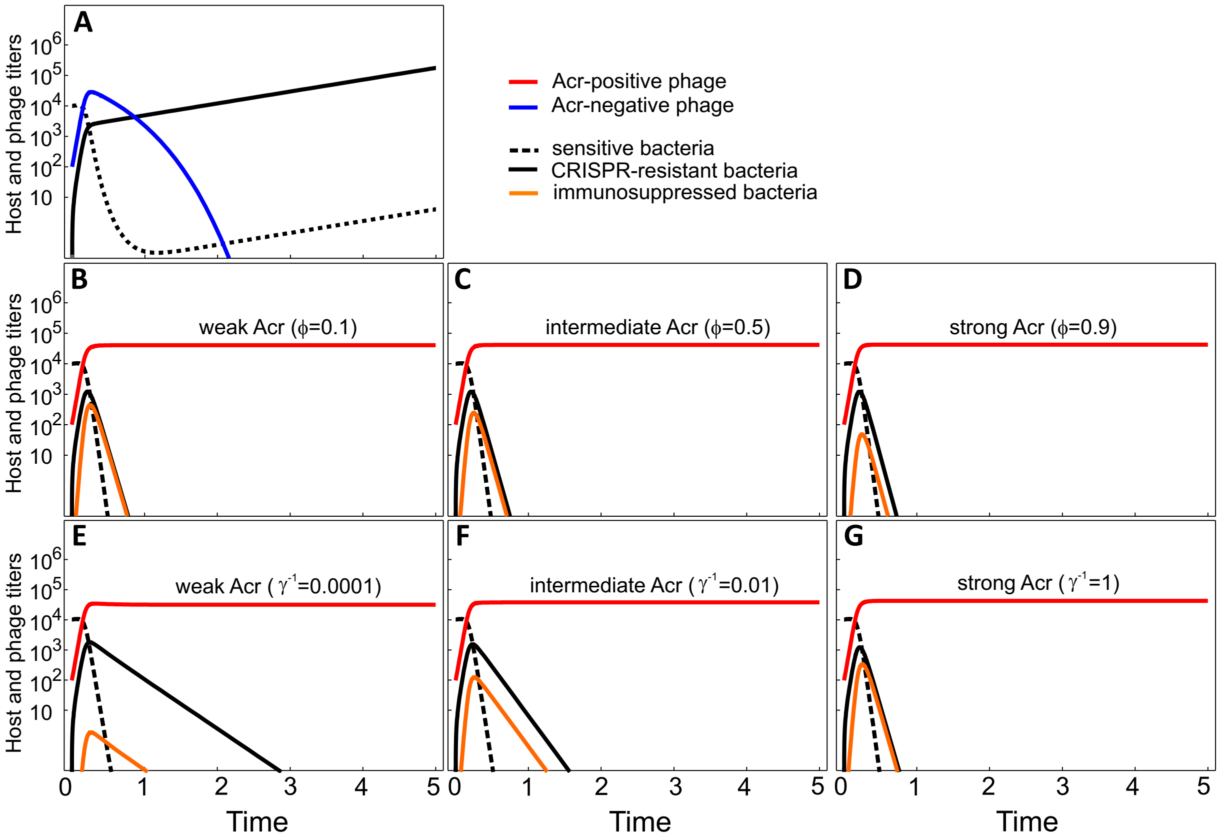

Figure S5. Simulations of phage/host evolutionary and population dynamics during individual infections. Related to Figure 5.

(**A**) Infection dynamics and evolution of CRISPR-resistance (solid black line) upon infection of initially sensitive hosts (dashed line) with 100 Acr-negative phages (blue line), or (**B-G**) upon infection with 100 Acr-positive phages (red lines), with different parameter values for ϕ and γ as follows: (B) $\phi = 0.1$ and $\gamma^{-1} = 0.1$, (C) $\phi = 0.5$ and $\gamma^{-1} = 0.1$, (D) $\phi = 0.9$ and $\gamma^{-1} = 0.1$, (E) $\phi = 0.3$ and $\gamma^{-1} = 0.0001$, (F) $\phi = 0.3$ and $\gamma^{-1} = 0.01$, (G) $\phi = 0.3$ and $\gamma^{-1} = 1$.

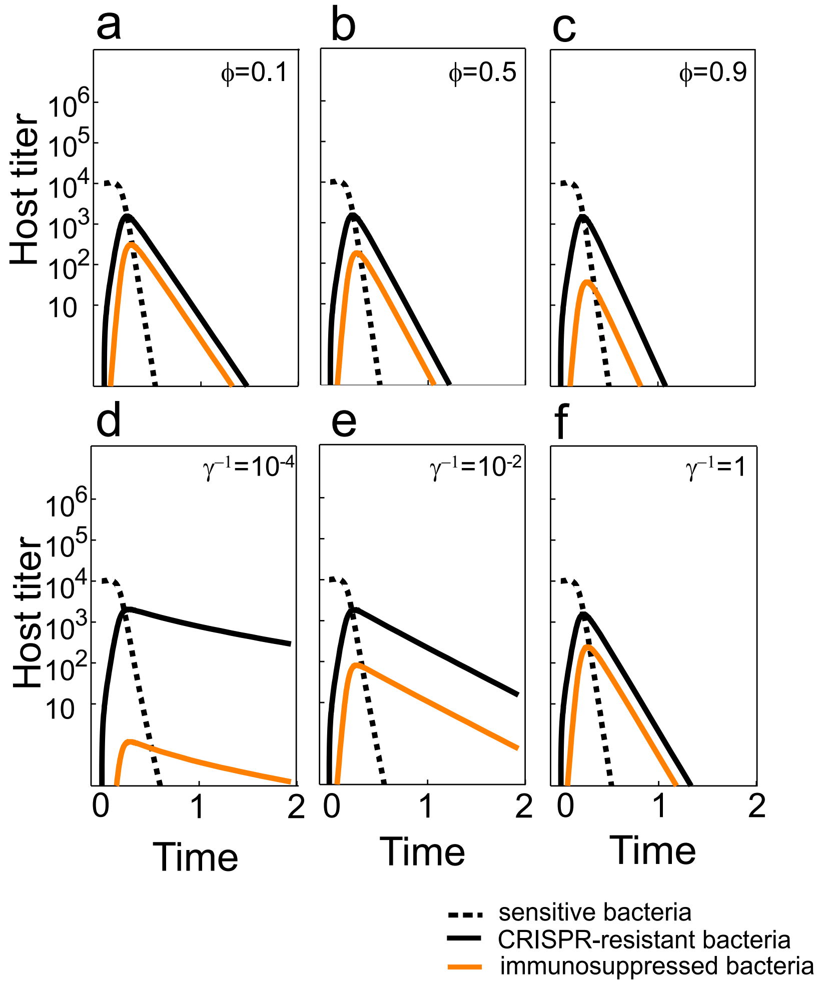

Figure S6. Simulations of host evolutionary and population dynamics during infections with mixed phage populations. Related to Figure 5.

Infection dynamics and evolution of CRISPR-resistance (solid black line) upon infection of initially sensitive hosts (dashed line) with an equal mix of Acr-negative and Acr-positive phage with different parameter values for ϕ and γ as follows: (a) $\phi = 0.1$ and $\gamma^{-1} = 0.1$, (b) $\phi = 0.5$ and $\gamma^{-1} = 0.1$, (c) $\phi = 0.9$ and $\gamma^{-1} = 0.1$, (d) $\phi = 0.3$ and $\gamma^{-1} = 0.0001$, (e) $\phi = 0.3$ and $\gamma^{-1} = 0.01$, (f) $\phi = 0.3$ and $\gamma^{-1} = 1$. Other parameter values: a = 0.001, A = 0.2, B = 5, $\rho = 0.5$.

Table S1. List of PAO1::*spycas9* indicator strains used to monitor individual concentrations of competing phages. Related to STAR Methods.

Indicator strain name (PAO1*::spycas9* + pJB1)	Phage sensitivity	Spacer sequence inserted into pJB1
Positive control	Resistant to all phages	5'-GTCTGCGCGGCGAGATATACG
Anti-AcrIF1	Resistant to DMS3*vir-acrIF1* only	5'-GTCGTAAACGGCAAAAGCGTT
Anti-AcrIF4	Resistant to DMS3*vir-acrIF4* only	5'-GTAAGCAACGGGTGGCAGTGG
Anti-Acr(-)	Resistant to DMS3*vir* only	5'-ATTTGGTGGCGACGCCGGCCG
Negative control	Sensitive to all phages	None

Table S2. List of primers used in this study. Related to STAR Methods.

Primer name	Sequence (5' – 3')
CRISPR array 1_SeqF	GGCGCTGGAGCCCTTGGGGCTTGG
CRISPR array 1_SeqR	GCGGCTGCCGGTGGTAGCGGGTG
CRISPR array 2_SeqF	GCTCGACTACTACAACGTCCGGC
CRISPR array 2_SeqR	GGGTTTCTGGCGGGAAAAACTCGG
CRISPR array 1_F	CTAAGCCTTGTACGAAGTCTC
CRISPR array 1_R	CGCCGAAGGCCAGCGCGCCGGTG
CRISPR array 2_F	GCCGTCCAGAAGTCACCACCCG
CRISPR array 2_R	TCAGCAAGTTACGAGACCTCG
DMS3*vir*_F	ACCTGAGCGAGGATCAATGG
DMS3*vir*_R	CGCGGCGAACCTTCTG
DMS3*vir-acrIF1*_F	CGAAAATGGCAGCAAAATCAA
DMS3*vir-acrIF1*_R	CCAACGCTTTTGCCGTTT
DMS3*vir-acrIF4*_F	GTGGCGCCCTCCATCAT
DMS3*vir-acrIF4*_R	GCAAGCCGAAGTAACCATTCTC
Δ*acr*-Up_F	TACCCATGGGATCTGATAAGAATTCGAGCTATCCGTCTGCGCGGCGAGATA
Δ*acr*-Up_R	CGTGTAGCGCGTTTGCGGGCGGATCAGGTGAAGGCACAGTGTGCCGCTTGT C
Δ*acr*-Down_F	TCACCTGATCCGCCCGCAAAC
Δ*acr*-Down_R	GACGGCCAGTGCCAAGCTTGCATGCCTGCACATTCGAAATCGAGGAAGCG GC
