# Supplementary material for: Exploitation of the Cooperative Behaviors of Anti-CRISPR Phages
Source: Cell Host Microbe. 2020 Feb 12;27(2):189–198.e6. doi: 10.1016/j.chom.2019.12.004 (PMC7013381; doi:10.1016/j.chom.2019.12.004)
Supplement: Document S2. Article plus Supplemental Information [file mmc2.pdf]

Article

Cell Host & Microbe

Exploitation of the Cooperative Behaviors of Anti-CRISPR Phages

Graphical Abstract

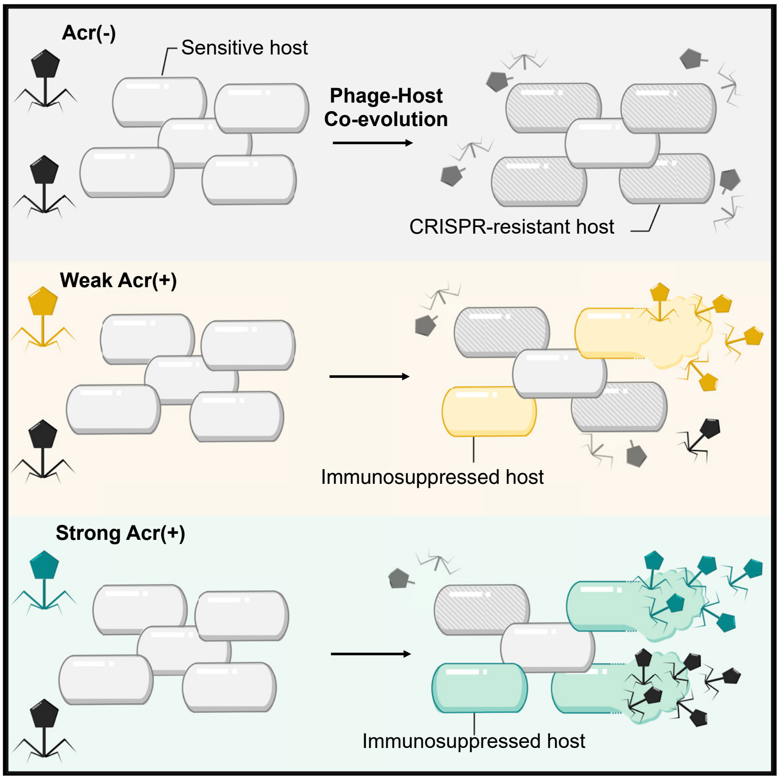

Authors

Anne Chevallereau, Sean Meaden, Olivier Fradet, ..., Sylvain Gandon, Stineke van Houte, Edze R. Westra

Correspondence

a.chevallereau@exeter.ac.uk (A.C.),
e.r.westra@exeter.ac.uk (E.R.W.)

In Brief

Chevallereau et al. find that phages encoding anti-CRISPR proteins (Acrs) protect the larger phage community, including Acr-negative phages, from bacterial CRISPR-Cas immunity by immunosuppressing resistant cells and limiting immune adaptation. Because they do not allow for such exploitation, low-efficiency Acrs provide a greater advantage during competition with Acr-negative phages.

Highlights

- Acr-positive phages limit evolution of CRISPR resistance during clonal and mixed infections
- Acr-positive phages provide benefits to Acr-negative phages present in the community
- Strong Acr help Acr-negative phages to amplify on immunosuppressed CRISPR-resistant cells
- Weaker Acr provides larger advantages during competition with Acr-negative phages

Chevallereau et al., 2020, Cell Host & Microbe *27*, 189–198
February 12, 2020 © 2019 The Authors. Published by Elsevier Inc.
https://doi.org/10.1016/j.chom.2019.12.004

CellPress

Exploitation of the Cooperative Behaviors of Anti-CRISPR Phages

Anne Chevallereau,[1,5,*] Sean Meaden,[1] Olivier Fradet,[1] Mariann Landsberger,[1,4] Alice Maestri,[1] Ambarish Biswas,[2] Sylvain Gandon,[3] Stineke van Houte,[1] and Edze R. Westra[1,*]
[1]ESI, Biosciences, University of Exeter, Cornwall Campus, Penryn TR10 9EZ, UK
[2]Department of Microbiology and Immunology, University of Otago, PO Box 56, Dunedin 9054, New Zealand
[3]CEFE, Université de Montpellier, CNRS, EPHE, IRD, Université Paul Valéry Montpellier 3, Montpellier, France
[4]Present address: MRC Laboratory for Molecular Cell Biology, University College London, London, UK
[5]Lead Contact
*Correspondence: a.chevallereau@exeter.ac.uk (A.C.), e.r.westra@exeter.ac.uk (E.R.W.)
https://doi.org/10.1016/j.chom.2019.12.004

SUMMARY

Bacteriophages encoding anti-CRISPR proteins (Acrs) must cooperate to overcome phage resistance mediated by the bacterial immune system CRISPR-Cas, where the first phage blocks CRISPR-Cas immunity in order to allow a second Acr phage to successfully replicate. However, in nature, bacteria are frequently not pre-immunized, and phage populations are often not clonal, exhibiting variations in Acr presence and strength. We explored how interactions between Acr phages and initially sensitive bacteria evolve, both in the presence and absence of competing phages lacking Acrs. We find that Acr phages benefit "Acr-negative" phages by limiting the evolution of CRISPR-based resistance and helping Acr-negative phages to replicate on resistant host sub-populations. These benefits depend on the strength of CRISPR-Cas inhibitors and result in strong Acrs providing smaller fitness advantages than weaker ones when Acr phages compete with Acr-negative phages. These results indicate that different Acr types shape the evolutionary dynamics and social interactions of phage populations in natural communities.

INTRODUCTION

Viruses of bacteria (phages) are generally acknowledged to be the most abundant entities on Earth and are thought to play a major role in shaping microbial ecology and evolution (Koskella and Brockhurst, 2014). CRISPR-Cas (clustered regularly interspaced short palindromic repeats; CRISPR-associated) adaptive immune systems are widespread mechanisms that can protect bacteria against phage infections (Hille et al., 2018; Barrangou and Horvath, 2017; Koonin et al., 2017). These adaptive immune systems insert short sequences derived from invading phage genomes (spacers) into CRISPR loci on the host genome. Transcription of these loci produces small RNAs that associate with Cas proteins to form a "surveillance" complex that enables the bacterium to detect and cleave infecting phage genomes carrying the cognate sequence (protospacer). Evolution of CRISPR-based resistance can drive rapid phage extinction (Van Houte et al., 2016), and in the face of this immune system, some phages have therefore evolved to produce anti-CRISPR proteins (Acrs) that block the CRISPR surveillance complex or the effector nucleases (Stanley and Maxwell, 2018). These Acrs naturally vary in their potency to suppress the host immune system, with some allowing phages to efficiently bypass the CRISPR-Cas system (strong Acr) while others are weaker inhibitors (Borges et al., 2018; Landsberger et al., 2018). Crucially, in the case of the type I-F CRISPR-Cas system, it has been shown that Acr phages need to work together to infect hosts that are already CRISPR resistant (Borges et al., 2018; Landsberger et al., 2018). Specifically, while many infections of CRISPR-resistant hosts fail initially, Acr phages leave behind an immunosuppressed cell, which is presumably due to the expression of the *acr* gene prior to the degradation of the phage genome mediated by Cas nucleases (Stanley et al., 2019). These immunosuppressed hosts can then be successfully exploited upon re-infection by other Acr phages, thereby supporting the amplification of clonal Acr phage populations (Borges et al., 2018; Landsberger et al., 2018). This results in ecological dynamics where a density threshold needs to be reached in order for the Acr phage population to amplify but their evolutionary dynamics remain unexplored. Notably, bacteria will often not be naturally pre-immunized but instead they will often be naive (i.e., not carrying a targeting spacer) or primed (i.e., carrying a mismatched spacer). Therefore, their ability to evolve CRISPR resistance in the presence of Acr phages is likely to be a critical factor that shapes phage-host interactions. Despite being the most likely scenario in nature, interactions of Acr phages with initially sensitive bacteria have not yet been studied, and how these *acr* genes influence the evolutionary dynamics of bacterial hosts is unknown. Moreover, with Acr delivery viewed as a "public good," it has been speculated that Acr-mediated immunosuppression could also protect other mobile genetic elements (MGEs) against CRISPR-Cas immunity (Nussenzweig and Marraffini, 2018). In this work, we investigate if and how phages without Acr activity could "cheat" on Acr phages, and how this impacts the evolutionary and population dynamics of the host and phages.

© 2019 The Authors. Published by Elsevier Inc.
This is an open access article under the CC BY license (http://creativecommons.org/licenses/by/4.0/).

RESULTS

Acr Phages Limit the Acquisition of CRISPR Resistance during Clonal Infection

To explore these questions, we first studied the individual interactions between phages with (Acr-positive) or without (Acr-negative) Acr activity and their host. We used the model system of *Pseudomonas aeruginosa* wild-type (WT) strain PA14 that is initially sensitive to the non-lysogenic phage DMS3*vir*, which naturally encodes an Acr protein (AcrIE3) that is inactive against the type I-F CRISPR-Cas system carried by PA14 (therefore referred to as "Acr-negative phage"). As "Acr-positive phages", we used isogenic versions of DMS3*vir* that carry allelic replacements of the *acrIE3* gene with *acrIF1* or *acrIF4*, which encode respectively strong and weak inhibitors of the type I-F CRISPR-Cas system of PA14 (Landsberger et al., 2018; Borges et al., 2018).

WT *P. aeruginosa* PA14 or isogenic CRISPR knockout (CRISPR-KO) strains were individually infected with phages and serially passaged for 3 days. In both experiments, we observed that the initially low phage-bacteria ratio (multiplicity of infection [MOI]) rapidly reached high levels ($\sim 10^3$) at 1 day post-infection (dpi) and subsequently declined (Figures 1A and 1B). These variations have important evolutionary consequences since higher MOI tends to select bacteria that acquire surface-based resistance over CRISPR-based resistance, while low MOI favors the evolution of CRISPR-based resistance (Westra et al., 2015). Interestingly, the population dynamics of Acr-positive phages were not affected by the presence of a functional CRISPR-Cas system in the host population (Figures 1A and 1B), whereas Acr-negative phages were rapidly driven to extinction by WT bacteria (Figure 1B). This is because WT bacteria rapidly evolved CRISPR-based resistance against Acr-negative phages under these experimental conditions, as described previously (Westra et al., 2015; Van Houte et al., 2016; Morley et al., 2017) and confirmed by deep sequencing analysis of the host CRISPR loci on day 3 post-infection (Figures 1C and 1D). Of the two CRISPR arrays carried by WT PA14, CRISPR 2 contains a spacer having 5 mismatches with gene 42 of DMS3*vir*, which allows the "primed" acquisition of new targeting spacers into both CRISPR arrays. As is typical of type I-F primed acquisition, the phage sequences targeted by these new spacers (i.e., protospacers) clustered around DMS3*vir* gene 42, with upstream and downstream protospacers located on the positive and negative strands, respectively (Figure 1E) (Westra et al., 2015). In contrast, very low frequencies of "primed" spacer acquisition were detected following infection with Acr-positive phages (Figures 1C–1E). As a result, the benefits of carrying a functional adaptive CRISPR-Cas system were lost when bacteria were exposed to Acr-positive phages, compared to Acr-negative phages, even when the Acr was a weak inhibitor of CRISPR-Cas (Figure 1F). Interestingly, our data showed that the two Acr variants enhanced phage survival to comparable levels (Figure 1B), as they both efficiently reduced the proportion of CRISPR-resistant hosts that evolved in the population (Figure 1G). These data suggest that Acr-positive phages may benefit related Acr-negative phages in the community, not only by immunosuppressing the CRISPR-resistant cells in the host population, as previously suggested (Nussenzweig and Marraffini, 2018), but also by limiting the evolution of this CRISPR-resistant host sub-population in the first place.

Acr-Negative Phages Benefit from the Presence of Acr-Positive Phages

To explore this hypothesis, we generated mixed phage populations (50:50 mix of Acr negative:Acr positive with strong or weak Acr) to infect WT PA14 and monitored the relative frequencies of each phage type for 3 days. As a control, we first verified that in the absence of a functional CRISPR-Cas system, the presence of Acr-positive phages had no impact on the amplification of Acr-negative phages at 3 dpi (Figures 2A and S2A). Moreover, Acr-negative and Acr-positive phages had equal fitness to one another (Figure 2B) and to a deletion mutant lacking the full *acr* operon (Δ*acr*) (Figure S1), suggesting that encoding an *acr* operon is cost free under these experimental conditions. Interestingly, when the host population carried a functional CRISPR-Cas system, the presence of Acr-positive phages had a large impact on Acr-negative phages, enabling them to avoid extinction after 3 days (Figure 2C). Looking at the compositions of phage populations over time, they shifted toward an increase in the proportion of Acr-positive phages, which means that carrying a functional *acr* gene that blocks the host immune system increases the relative fitness of the phage (Figure 2D). Surprisingly, while this increase in relative fitness was moderate for phages with the strong Acr, it was much larger for phages encoding the weak Acr (Figure 2D). These data therefore indicate that encoding a strong Acr activity is less advantageous than a weak one for Acr phages, when they compete with Acr-negative phages.

Phages with Strong Acr Reduce the Evolution of CRISPR Resistance against Acr-Negative Phages

We wondered how Acr-positive phages enabled the survival of Acr-negative phages and why this effect depended on Acr strength. We hypothesized that it could result from either a reduction in the evolution of CRISPR resistance (see Figure 1), the immunosuppression of CRISPR-resistant cells (Nussenzweig and Marraffini, 2018), or both. To explore the first hypothesis, we analyzed the evolution of CRISPR resistance over time when hosts were infected with the mixed phage population. This showed that, compared to infections with Acr-negative phages alone, evolution of CRISPR-resistance was suppressed in the presence of mixed phage populations and more strongly so when the Acr was strong (Figures 3A and 3B). This could be due to a stronger reduction of spacer acquisition (Vorontsova et al., 2015) or to a more rapid depletion of hosts that evolved resistance by phages with strong Acrs. In support of this latter hypothesis, we found that the strong Acr phages caused a stronger selection against CRISPR-resistant bacteria with high levels of CRISPR resistance (carrying 2 spacers "BIM-2sp") during competition with surface mutant (Sm) bacteria that lack the phage receptor and are therefore equally resistant against Acr-negative and Acr-positive phages (Figure 3C). Importantly, the MOI pressure chosen in this experiment falls within the range of MOIs observed in Figures 1A and 1B. Collectively, these data show that Acr-positive phages can indirectly enhance the survival of Acr-negative phages by limiting the evolution of CRISPR resistance, but they do not exclude the possibility that generation of immunosuppressed cells also contributes to this survival effect.

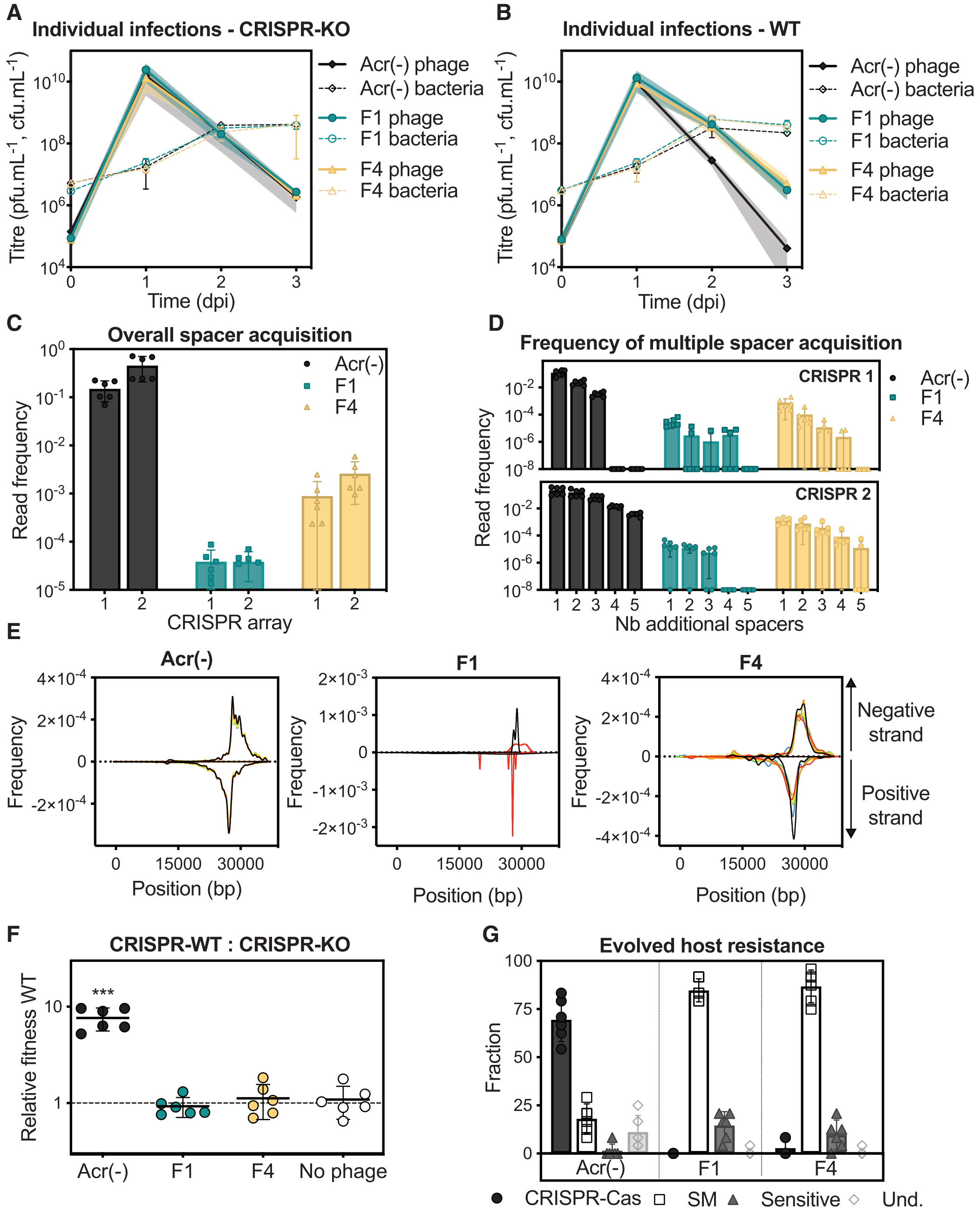

Figure 1. Impact of *acr* Genes on Phage Population Dynamics and Evolution of CRISPR Resistance during Infection of the Initially Sensitive WT Host Population

(A and B) Phage (solid lines) and bacterial (dashed lines) populations dynamics upon individual infections of the CRISPR-KO (A) or the WT host by Acr-negative or Acr-positive phages with different Acr (B).

(C) Frequency of spacer acquisition in CRISPR arrays 1 and 2 at 3 dpi in the evolved WT PA14 populations, as determined by deep sequencing.

(D) Frequencies of reads containing 1, 2, 3, 4, or 5 additional spacers.

(E) Protospacer distributions. Newly acquired spacers were extracted from read sequences and corresponding protospacers were mapped back to phage genomes, on positive and negative strands. Observed distributions are consistent with primed spacer acquisition.

(F) Relative fitness of WT PA14 and CRISPR-KO strains at 3 dpi in the presence or absence of indicated phages at an initial MOI of 0.01. One-sample t tests indicate significant differences from 1 for “Acr(–)” ($p = 0.0004$ and $t_5 = 8.3$) and no significant differences for F1 ($p = 0.42$ and $t_5 = 0.87$), F4 ($p = 0.52$ and $t_5 = 0.52$), and No phage control ($p = 0.61$ and $t_5 = 0.54$).

(G) Distribution of phage-resistance mechanisms that evolved at 3 dpi (based on analysis of 24 clones per replicate). CRISPR-Cas, detection of additional spacers assessed by PCR; Sm, surface mutant; Und., undetermined. These are clones for which the results were inconclusive.

In all panels, data shown are the mean of 6 biological replicates per treatment. Shaded areas and error bars represent 95% confidence intervals (CIs). F1, DMS3*vir-acrIF1*; F4, DMS3*vir-acrIF4*; and Acr(–), DMS3*vir*.

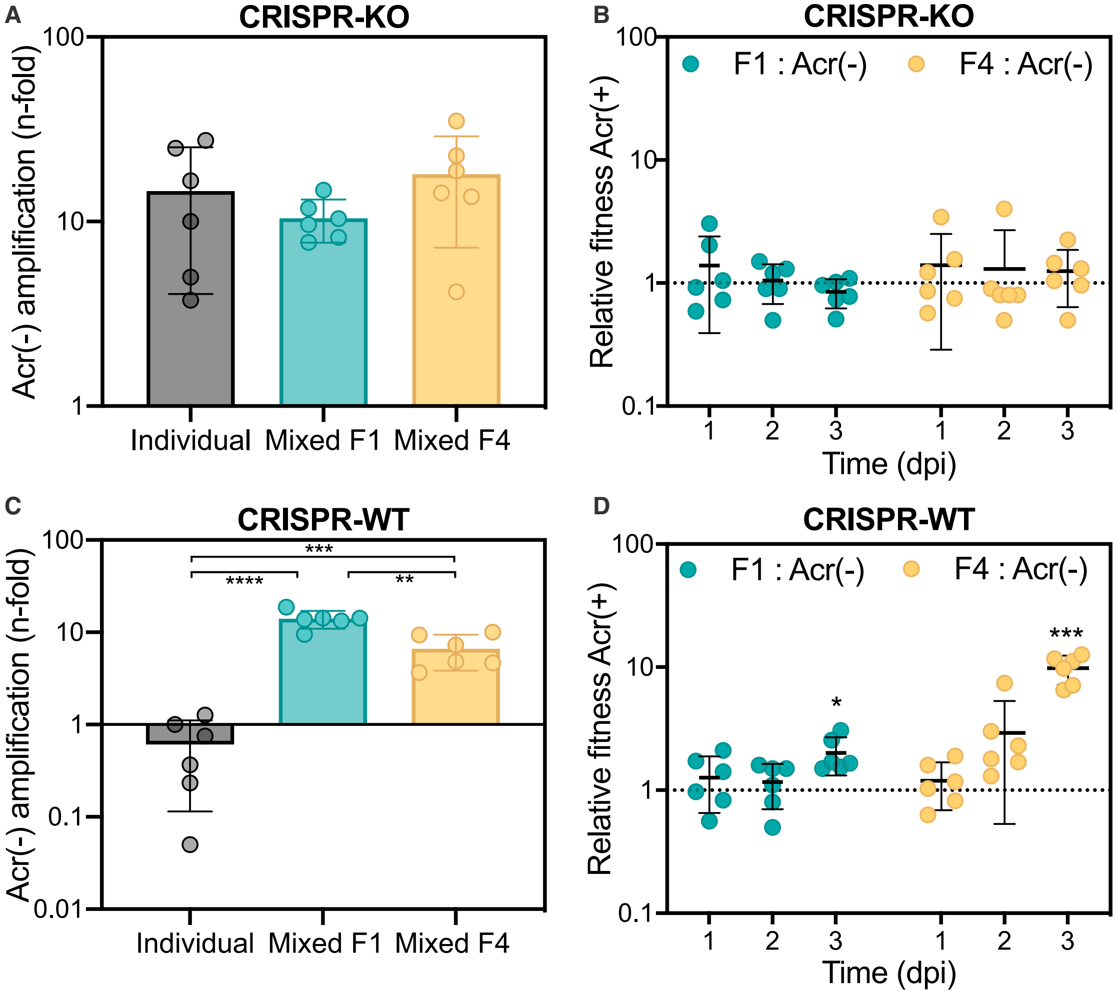

Figure 2. Acr-Positive Phages Benefit Acr-Negative Phages

(A) Amplification of Acr-negative phage population between T = 0 and 3 dpi in the absence (individual) or in the presence (mixed) of indicated Acr-positive phages.

(B) Relative fitness of Acr-positive phages during competition on the CRISPR-KO strain. See also Figure S1.

(C) Amplification of Acr-negative phage population between T = 0 and 3 dpi in the absence (individual) or in the presence (mixed) of indicated Acr-positive phages.

(D) Relative fitness of Acr-positive phages during competition on the WT strain. Asterisks indicate statistically significant differences. Two-tailed t tests (C) individual versus mixed F1: $p < 0.0001$ and $t_{10} = 11$; individual versus mixed F4: $p = 0.0003$ and $t_{10} = 5.49$; and mixed F1 versus mixed F4: $p = 0.0011$ and $t_{10} = 4.56$. One-tailed t tests at 3 dpi (D) F1:Acr(−): $p = 0.013$ and $t_5 = 3.78$ and F4:Acr(−): $p = 0.0003$ and $t_5 = 8.63$.

In all panels, data shown are the mean of 6 biological replicates per treatment and error bars represent a 95% CI.

The Presence of Strong Acr Phages Enables Acr-Negative Phages to Infect CRISPR-Resistant Hosts

To test this second hypothesis, we examined whether Acr-negative phages were able to amplify on CRISPR-resistant hosts in the presence of Acr-positive phages and how this depended on the level of host resistance. Acr-positive phages can amplify on CRISPR-resistant hosts but only under conditions where sequential infections are likely to occur, i.e., when the MOI is above a certain value. Moreover, this critical MOI threshold depends both on the strength of the Acr and on the level of host resistance, which increases with the number of targeting spacers (Figures S2B and S2C) (Landsberger et al., 2018). In contrast, Acr-negative phages on their own can never reproduce on a pre-immunized host population (Figures S2B–S2D), unless they carry "escape" mutations in their protospacer (Figures S2C–S2E, pink circled dots), but these are less likely to arise when the host carries multiple spacers (BIM-2sp, Figure S2D).

We therefore infected pre-immunized bacteria carrying one (BIM-1sp) or two (BIM-2sp) targeting spacers with 50:50 mixtures of Acr-positive and Acr-negative phages. We observed that Acr-negative phages became able to multiply on CRISPR-resistant hosts in the presence of phages encoding the strong Acr when the initial MOI was above 0.1 (Figures 4A and 4B), indicating that such exploitation of strong Acr may occur when sequential infections are likely. In contrast, this was never observed in the presence of phages encoding a weak Acr (Figures 4C and 4D), even when the MOI was high enough to allow amplification of the Acr-positive phages (i.e., ensuring that sequential infections, or even co-infections, occur in these conditions). We verified that all Acr-negative phages that amplified

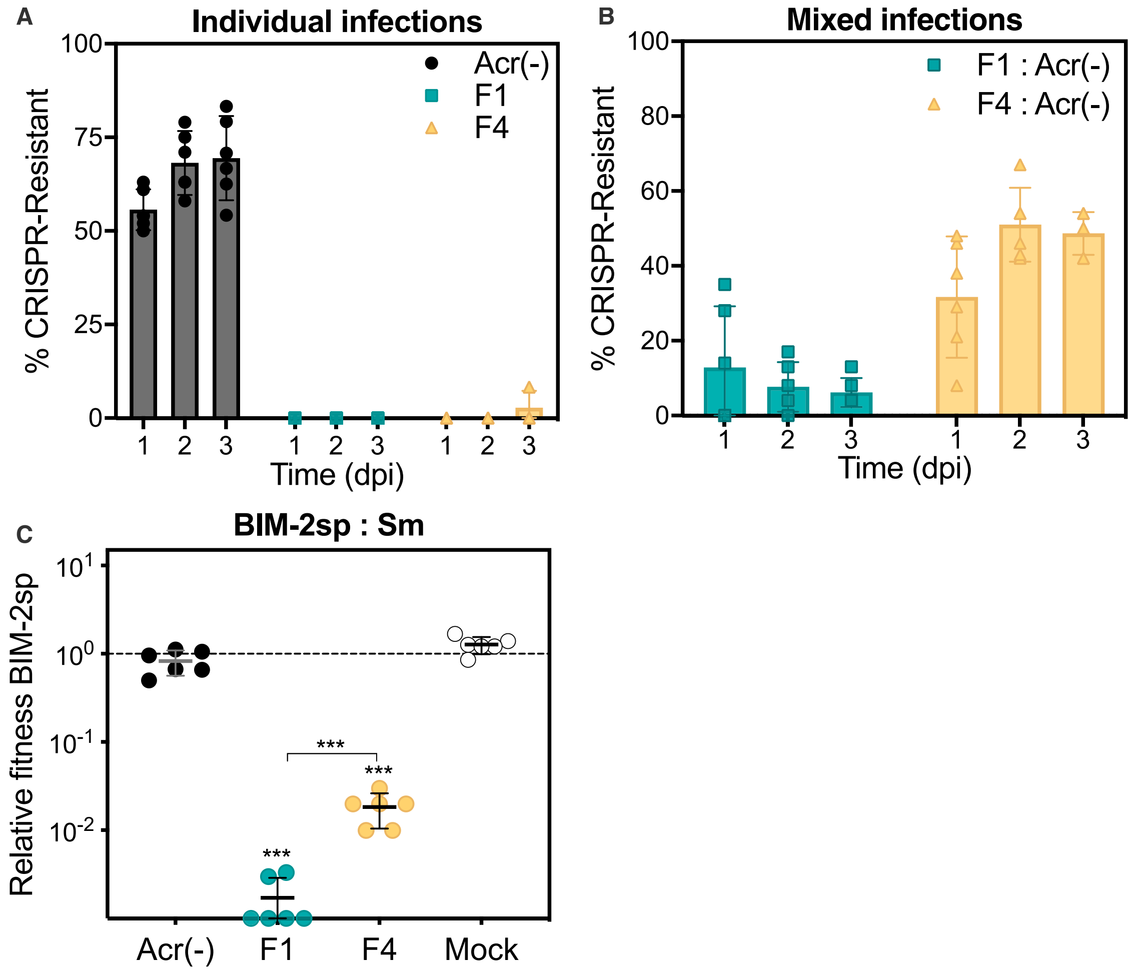

Figure 3. Presence of Strong Acr Phages Strongly Reduces the Evolution of CRISPR Resistance

(A and B) Proportion of WT hosts evolving CRISPR-based resistance over time upon individual (A) or mixed infections (B) (n = 144; 24 clones tested in each of 6 independent infections).

(C) Relative fitness of bacteria with CRISPR resistance (BIM-2sp, 2 targeting spacers) and surface mutant (Sm) in the presence of indicated phages (MOI ~ 25). Significant differences from 1 (one-tailed t tests: F1, $p < 0.0001$ and $t_5 = 2189$ and F4, $p < 0.0001$ and $t_5 = 319$) or in-between values (two-tailed t test: F1 versus F4, $p = 0.0003$ and $t_{10} = 5.35$) are indicated.

In all panels, data shown are the mean of 6 biological replicates per treatment and error bars represent a 95% CI.

after 24 h in the presence of the strong Acr phage (green-circled dots, Figures 4A and 4B) were still sensitive to CRISPR immunity (Figures 4E and 4F) and had not acquired escape point mutations (Figures S3A and S3C), whereas those that amplified in the presence of the weak Acr phage (pink circled dots, Figure 4C) had acquired escape mutations that made them insensitive to CRISPR targeting (Figures 4E and S3B). Even though they did not amplify, WT Acr-negative phages could still benefit from the presence of weak Acr phages, as demonstrated by their reduced rate of decline (mild protective effect, compare black lines in Figures S2D and 4D). Altogether, these results show that Acr-negative phages may benefit from the presence of Acr-positive phages through two mechanisms. First, the Acr-positive phages can indirectly protect Acr-negative phages from CRISPR degradation by limiting the evolution of CRISPR-resistance, and second, they can directly facilitate the replication of Acr-negative phages by producing immunosuppressed hosts.

A Model to Understand How Variations in Acr Biochemistry Shape the Population and Evolutionary Dynamics of Bacteria and Phages

Next, to understand why strong and weak Acrs differ in the benefits they share with the wider phage community, we refined our previously published mathematical model (Landsberger et al., 2018), which allows us to break down different components of Acr activity and track the dynamics of each member of the community (i.e., Acr-negative and Acr-positive phages as well as sensitive, immunosuppressed, and CRISPR-resistant bacteria). To match with our experimental system, this model assumes that bacteria are initially sensitive (W) but can evolve CRISPR-based resistance upon phage infection (Figure 5A). Infection of a CRISPR-resistant bacterium (R) by an Acr-positive phage can lead either to cell lysis (V) with probability φ (which leads to depletion of the CRISPR-resistant sub-population) or result in a failed infection that leaves the host in an immunosuppressed state (S)

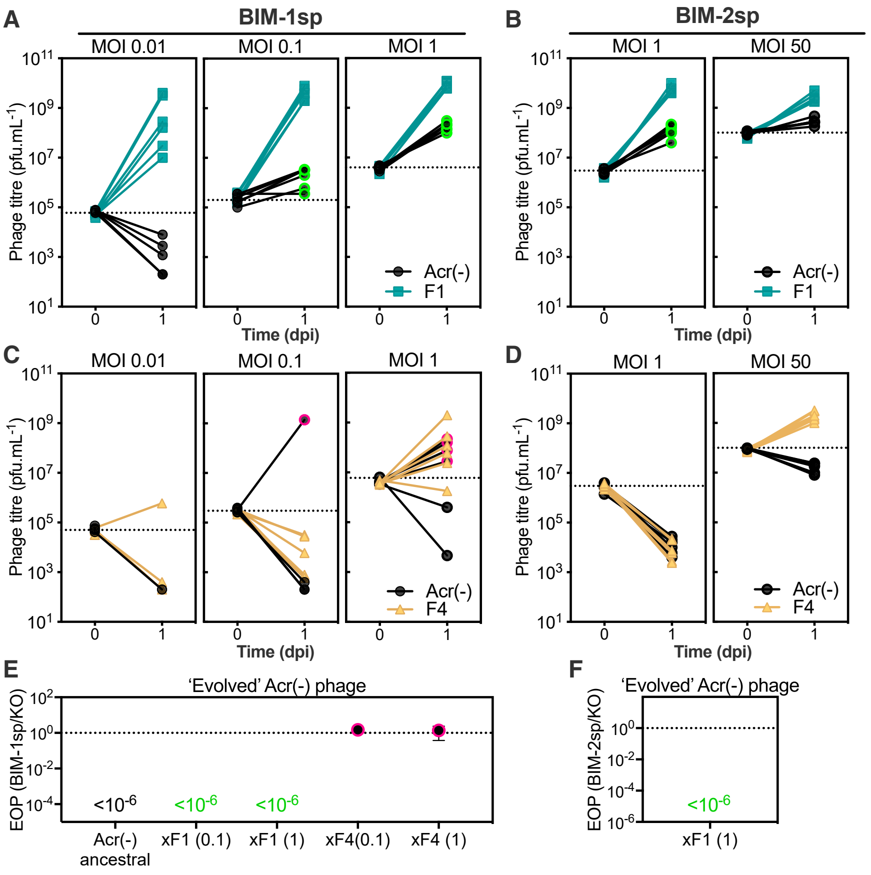

Figure 4. The Presence of Strong Acrs Enables Acr-Negative Phages to Replicate on CRISPR-Resistant Hosts

Amplification of Acr-negative phages in the presence of Acr-positive phages encoding (A and B) strong AcrIF1 or (C and D) weak AcrIF4 upon infection of the CRISPR-resistant host carrying (1) (A and C) or (2) (B and D) spacers targeting both phages. As amplification of Acr-positive phages is MOI dependent, several initial MOIs were tested as indicated at the top of the graphs. Acr-negative phages that amplified after 24 h co-culture with F1 (green circled dots) or with F4 (pink circled dots) were isolated and the ability of these "evolved" Acr-negative phages to infect (E) BIM-1sp or (F) BIM-2sp hosts on their own was assessed with EOP assays. Graphs show individual data (A–D) or means (E and F) from 6 independent biological replicates. Error bars indicate a 95% CI.

See also Figures S2 and S3.

(which provides an opportunity for other phages to exploit immunosuppressed cells). Immunosuppressed cells revert back to the resistant state after a certain duration γ^{-1}. Hence, φ and γ^{-1} both measure the "strength" of an Acr but likely reflect different biochemical properties, such as the affinity for the Cas protein it targets and the stability of the Acr itself or that of the Acr-Cas interaction. Experimental estimates of the efficiency of centers of infection (ECOI), as a proxy for φ, show that the probability to lyse a CRISPR-resistant host is higher for the AcrIF1 phage (Figure S4). Therefore, this suggests that the higher strength of AcrIF1 is (at least partly) explained by a higher value of φ. To further explore the respective influences of φ and γ^{-1} on the phage and host population dynamics (if any) during infection of initially sensitive bacteria, we can vary both parameters independently in our simulations (see STAR Methods for a full description of the model). As a control, we first confirmed that model predictions during infections with clonal phage populations were consistent with our empirical data. Indeed, the model predicts that individual infection of initially sensitive bacteria with Acr-negative phages leads to rapid phage extinction due to the evolution of CRISPR-based resistance (Figure S5A), whereas Acr-positive phages avoid extinction across a large range of φ and γ^{-1} values (Figures S5B–S5G consistent with Figures 1A and 1B, i.e., no differences between Acr variants in this context). Next, we explored the effects of φ and γ^{-1} during infections of WT bacteria with mixed phage populations and found that both Acr-positive and Acr-negative phages avoid extinction in this context (Figure 5B).

Mathematical Modeling Predicts that Acrs Differ in Their Ability to Generate Lasting Immunosuppression

Given that phages encoding AcrIF1 have higher ECOI and efficiency of plaquing (EOP) values than those encoding AcrIF4 (Figure S4) (Landsberger et al., 2018), we first explored how manipulating φ impacts the bacteria and phage population dynamics. Interestingly, we found that increasing the value of φ resulted in an increased fitness advantage of the Acr-positive phage over the Acr-negative phages (Figure 5C). This intuitively makes sense since a high value of φ means a reduced frequency of failed infections (and therefore a lower proportion of immunosuppressed cells), which limits the opportunities for Acr-negative phages to reproduce. However, our experiments have shown that AcrIF4-phages (with the lowest φ value) had the highest fitness advantage over Acr-negative phages (Figure 2D). Therefore, the manipulation of φ alone, as a simulation of varying Acr strength, cannot explain our experimental data. By contrast, when we manipulated the value of γ^{-1}, we found that a longer-lasting immunosuppression resulted in a smaller fitness advantage during direct competitions with Acr-negative phages (Figure 5D). Hence, assuming that AcrIF1-phages not only lyse CRISPR-resistant cells more efficiently (i.e., higher value of φ) but also induce longer periods of immunosuppression when infections fail (i.e., a higher value of γ^{-1}) allows us to obtain a good fit between the experimental and simulation data. These high γ^{-1} values can explain why the AcrIF1 phages have a lower fitness advantage over Acr-negative phages compared to AcrIF4 phages (i.e., Figure 5D consistent with Figure 2D). In addition, when increasing the value of γ^{-1}, the model predicts that (1) the sub-population of CRISPR-resistant bacteria is depleted more rapidly (Figures S6D–S6F black lines, consistent with Figure 3B) and (2) greater numbers of immunosuppressed cells accumulate, which can be exploited by Acr-negative phages (Figures S6D–S6F orange lines, consistent

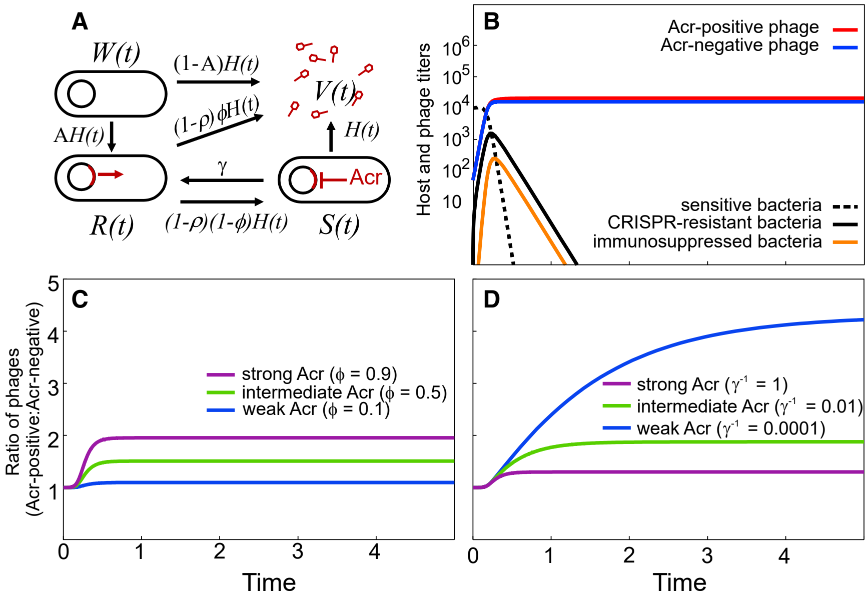

Figure 5. Modeling of Different Acr Strengths and Their Impact on Acr-Negative Phages

(A) Infection model of the Acr-positive phage (see details of the model in the STAR Methods). The parameter $H(t) = aV(t)$ refers to the rate at which bacteria are infected by free phage particles, A is the probability that bacteria acquire CRISPR-based resistance.

(B) Bacteria and phage population dynamics upon infection of initially sensitive hosts (dashed line) with an equal mix of 100 Acr-negative (blue line) and 100 Acr-positive phages (red line, $\varphi = 0.3$ and $\gamma^{-1} = 1$). Initially sensitive bacteria evolve CRISPR-resistance (solid black line), which in turn can become immunosuppressed upon infection by Acr-positive phages (orange line).

(C and D) Effect of φ (C) and γ^{-1} (D) on the ratio of Acr-positive and Acr-negative phages following infection of sensitive bacteria. Other parameter values: $a = 0.001$, $A = 0.2$, $B = 5$, and $\rho = 0.5$. See also Figures S4, S5, and S6.

with Figures 4A and 4B). Therefore, our results suggest that the observed differences between AcrIF1 and AcrIF4 may be explained by significant differences both in the ability to lyse resistant cells (φ in our model) and in the lifetime of immunosuppression (γ^{-1} in our model).

DISCUSSION

In summary, our results show that Acr-negative phages can benefit from the presence of Acr-positive phages to infect bacteria carrying type I-F CRISPR-Cas systems. In nature, the infection dynamics of a mixed population of Acr-positive and Acr-negative phages will also depend on the level of cross-reactivity of the CRISPR-Cas immune system against the competing phages. A recent study analyzing the CRISPR arrays from >700 *P. aeruginosa* genomes has shown that a given spacer usually provides such cross-reactivity, as it typically matches several viruses (e.g., 2.75 viruses on average), which, in general, are genetically close and co-occur in the same ecological niche (and hence are likely to compete for the same hosts), but some spacers have also been found to provide cross-reactive immunity against distantly related viruses (England et al., 2018).

Second, the model and data presented here also show that during competitive infections, the strength of the AcrIF protein determines to what extent its benefits are "shared" with other phages. Notably, the shared benefits provided by strong AcrIF proteins result from two effects. First, strong Acr phages deplete the pool of CRISPR-resistant hosts more rapidly than weak Acr phages, which is somewhat analogous to the way antibiotic-resistant bacteria can support growth of sensitive species by detoxifying the environment (Dugatkin et al., 2003, 2005; Medaney et al., 2016). Then, they enable Acr-negative phages to exploit CRISPR-resistant hosts through immunosuppression, whereas weak Acrs do not. The net result of these effects is that a strong Acr activity is less advantageous than a weak one for an Acr-positive phage when competing with Acr-negative phages. This may be important especially during the early evolution of new *acr* genes since those that are weak would invade the phage population more rapidly compared to strong ones. However, this could be a transient effect since in the longer term—when phages with different Acrs have emerged and compete against each other—weak Acrs no longer provide the greatest fitness benefit. Specifically, direct competition between phages with strong AcrIF1 and weak AcrIF4 showed that strong Acr phages were favored when the bacterial host population was already CRISPR-resistant, whereas the two Acr phages were found to be equally fit when the host population was initially phage sensitive (Figure 6). The fact that pre-existing CRISPR immunity is relatively rare, along with the non-transitivity of the competitive interactions, may therefore contribute to the coexistence of strong and weak Acr phages in nature.

Our mathematical prediction that AcrIF1 induces longer duration of immunosuppression (compared with AcrIF4) is consistent with data showing that it binds the Csy surveillance complex with higher affinity and slower off rates than AcrIF4 (Borges et al., 2018). This parameter γ^{-1} is critical to explain the smaller fitness benefit of AcrIF1 during competition with Acr-negative phages and effectively determines the potential for Acr "public goods" production: the greater the γ^{-1}, the more immunosuppressed cells are generated in the population and the greater the opportunity for other phages to exploit these immunosuppressed cells. This is analogous to the production of iron scavenging molecules, communication signals, and virulence factors, which are produced by few individuals but can benefit the whole population (Diggle et al., 2007; Griffin et al., 2004; Raymond et al., 2012). However, unlike these examples of altruistic cooperation, the production of Acr proteins surprisingly appears to be cost free in our system (Figure S1)—which may be due to the tight regulation of *acr* expression by the anti-CRISPR-associated protein Aca (Birkholz et al., 2019;

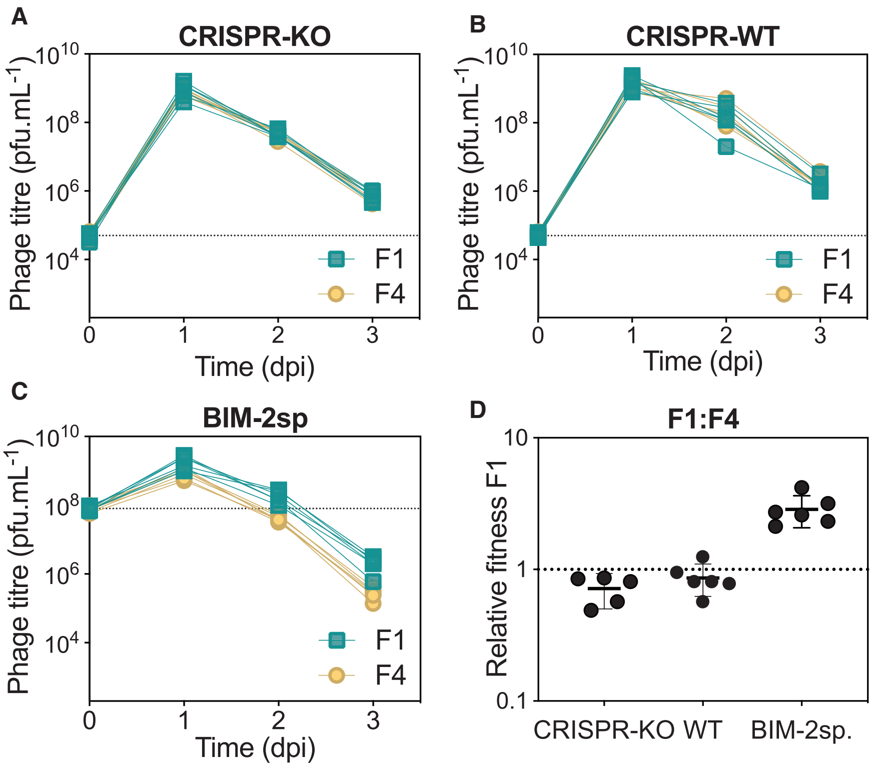

Figure 6. Phages with Strong Acr Do Not Have a Fitness Advantage over Phages with Weak Acr When Bacteria Are Initially Sensitive

(A–C) Population dynamics of weak and strong Acr-positive phages during mixed infection of (A) CRISPR-KO, (B) initially sensitive (WT), or (C) CRISPR-resistant (BIM-2sp) hosts.

(D) Fitness values of strong AcrIF1-phage relative to that of weak AcrIF4-phage were calculated on day 3.

Graphs show individual data (A)–(C) or means (D) from 6 independent biological replicates. Error bars indicate a 95% CI.

Stanley et al., 2019). That said, our experimental data do not rule out the possibility that encoding an *acr* operon carries a cost for phages in natural environments. Apart from direct costs due to necessary high levels of gene expression (Mahmoudabadi et al., 2017), there may also be a cost of opportunity as the overall genome size of the phage is constrained by the space in the phage capsid.

If carrying *acr* genes is indeed costly, then the stability of phage populations that evolve stronger "cooperative" Acr (high γ^{-1}) may be impaired by the invasion of Acr negative "cheats" that do not contribute to public good production while still sharing the benefits (Hamilton, 1964). The factor φ, on the other hand, may be best described as a selfish trait that reflects the probability that the first phage will neutralize CRISPR-Cas immunity and lyse the host. It therefore depends on the affinity of the Acr for the Cas interference complexes, and its value is constrained by the biochemical and biophysical properties of the Acr. Another way for the phage to evolve a stronger "selfish" Acr (high φ) is, for instance, to increase the production of Acr. However, this may be particularly costly because of limitations in the resource and energy availability in the cell. Note that in our model system, the strong and weak Acr phages are isogenic (except for the *acr* coding sequence), therefore the differences in φ are likely not due to differences in *acr* transcription. Overall, our study suggests that the composition of the phage population (i.e., whether clonal or mixed) is likely to be a key driver of differences in φ and γ^{-1}: higher φ and/or lower γ^{-1} may be favored during competition while lower φ and/or higher γ^{-1} will be favored by kin selection. In addition, one can speculate that mutations in *acr* genes may have pleiotropic effects (e.g., the ability to induce lasting immunosuppression might be a by-product of the strong inhibition of CRISPR-Cas surveillance complexes), and therefore φ and γ^{-1} cannot evolve independently (Dos Santos et al., 2018).

The effects of encoding strong or weak Acrs during competition with other MGEs will further depend on their transmission mode, such as their ability to transmit only horizontally (as in the current study) or both horizontally and vertically (as is the case for temperate phages and conjugative plasmids). For example, if the CRISPR-Cas system of the host targets the Acr-MGE, vertical transmission may be associated with selection for high γ^{-1} but weaken selection for φ. However, it is also possible that an active host immune system contributes to the fitness of a vertically transmitting Acr-MGE by providing protection against other parasitic MGEs, in which case weaker Acr activities might be positively selected. Future experiments will be critical to understand if and how interactions between the ecological context and the life history traits of MGEs impacts the evolution of strong or weak Acrs.

Finally, similar approaches aiming at understanding the ecological and evolutionary parameters that influence the selection for type II Acrs may be valuable for biotechnological applications. Interestingly, Acrs specific to Cas9 have also been reported to have variable strengths, e.g., $AcrIIC2_{Nme}$ is less potent than $AcrIIC1_{Nme}$ in inhibiting *Neisseria meningitidis* Nme1Cas9 (Thavalingam et al., 2019) and different AcrIIA proteins targeting *Streptococcus pyogenes* SpyCas9 demonstrated variable efficiencies in inhibiting CRISPR-based interference (CRISPRi) or activation (CRISPRa) in eukaryotic cells (Nakamura et al., 2019).

Future single cell analyses, as well as studies providing deeper insight into the biochemical bases of Acr-Cas interactions and accurate experimental measures of γ^{-1} and φ, will be necessary to further validate and refine the model presented here. This will be fundamental to fully understand the evolutionary drivers and consequences of *acr* genes with different strengths and their implications for the wider phage community and other MGEs that spread in the face of bacteria with CRISPR-Cas immune systems.

STAR★METHODS

Detailed methods are provided in the online version of this paper and include the following:

- KEY RESOURCES TABLE
- LEAD CONTACT AND MATERIALS AVAILABILITY
- EXPERIMENTAL MODEL AND SUBJECT DETAILS
  - Bacteria
  - Phages
- METHOD DETAILS
  - Individual Phage Infection Assays
  - Analysis of Evolved Phage Resistance
  - Bacterial Competition Experiments
  - Phage Competition Experiments
  - Fitness Costs Associated with *acr* Operon
  - Efficiency of Centre of Infection (ECOI) Assays
  - Mathematical Modelling
- QUANTIFICATION AND STATISTICAL ANALYSIS
- DATA AND CODE AVAILABILITY

SUPPLEMENTAL INFORMATION

Supplemental Information can be found online at https://doi.org/10.1016/j.chom.2019.12.004.

ACKNOWLEDGMENTS

The authors acknowledge the Centre for Genomic Research (University of Liverpool), in particular, R. Eccles, A. Lucaci, and R. Gregory for performing amplicon sequencing. The authors thank Dr. L. Zhang for help setting up qPCR analyses and Prof. A. Buckling for critical reading of the manuscript. This work was supported by grants from the European Research Council (ERC-STG-2016-714478 - EVOIMMECH), the Biotechnology and Biological Sciences Research Council (BB/N017412/1), the Wellcome Trust (109776/Z/15/Z), and the Natural Environment Research Council (NE/M018350/1), which were awarded to E.R.W. A.C. has received funding from the European Union's Horizon 2020 research and innovation program under the Marie Skłodowska-Curie grant agreement no. 834052.

AUTHOR CONTRIBUTIONS

Conceptualization, A.C. and E.R.W.; Methodology, A.C., S.M., M.L., and E.R.W.; Investigation, A.C., O.F., A.M., and S.v.H.; Formal Analysis, A.C., S.M, A.B., and S.G.; Writing – Original Draft, A.C.; Writing – Review & Editing, A.C. and E.R.W. with contributions from S.M., M.L., and S.G.; Supervision, E.R.W.; Funding Acquisition, A.C. and E.R.W.

DECLARATION OF INTERESTS

The authors declare no competing interests.

Received: September 25, 2019
Revised: November 13, 2019
Accepted: December 6, 2019
Published: December 31, 2019

REFERENCES

Barrangou, R., and Horvath, P. (2017). A decade of discovery: CRISPR functions and applications. Nat. Microbiol. *2*, 17092.

Birkholz, N., Fagerlund, R.D., Smith, L.M., Jackson, S.A., and Fineran, P.C. (2019). The autoregulator Aca2 mediates anti-CRISPR repression. Nucleic Acids Res. *47*, 9658–9665.

Biswas, A., Staals, R.H., Morales, S.E., Fineran, P.C., and Brown, C.M. (2016). CRISPRDetect: a flexible algorithm to define CRISPR arrays. BMC Genomics *17*, 356.

Bolyen, E., Rideout, J.R., Dillon, M.R., Bokulich, N.A., Abnet, C.C., Al-Ghalith, G.A., Alexander, H., Alm, E.J., Arumugam, M., Asnicar, F., et al. (2019). Reproducible, interactive, scalable and extensible microbiome data science using QIIME 2. Nat. Biotechnol. *37*, 852–857.

Borges, A.L., Zhang, J.Y., Rollins, M.F., Osuna, B.A., Wiedenheft, B., and Bondy-Denomy, J. (2018). Bacteriophage cooperation suppresses CRISPR-Cas3 and Cas9 immunity. Cell *174*, 917–925.e10.

Cady, K.C., Bondy-Denomy, J., Heussler, G.E., Davidson, A.R., and O'toole, G.A. (2012). The CRISPR/Cas adaptive immune system of Pseudomonas aeruginosa mediates resistance to naturally occurring and engineered phages. J. Bacteriol. *194*, 5728–5738.

Diggle, S.P., Griffin, A.S., Campbell, G.S., and West, S.A. (2007). Cooperation and conflict in quorum-sensing bacterial populations. Nature *450*, 411–414.

Dos Santos, M., Ghoul, M., and West, S.A. (2018). Pleiotropy, cooperation, and the social evolution of genetic architecture. PLoS Biol. *16*, e2006671.

Dugatkin, L.A., Perlin, M., and Atlas, R. (2003). The evolution of group-beneficial traits in the absence of between-group selection. J. Theor. Biol. *220*, 67–74.

Dugatkin, L.A., Perlin, M., Lucas, J.S., and Atlas, R. (2005). Group-beneficial traits, frequency-dependent selection and genotypic diversity: an antibiotic resistance paradigm. Proc. Biol. Sci. *272*, 79–83.

England, W.E., Kim, T., and Whitaker, R.J. (2018). Metapopulation structure of CRISPR-Cas immunity in *Pseudomonas aeruginosa* and its viruses. mSystems *3*.

Griffin, A.S., West, S.A., and Buckling, A. (2004). Cooperation and competition in pathogenic bacteria. Nature *430*, 1024–1027.

Hamilton, W.D. (1964). The genetical evolution of social behaviour. I. J. Theor. Biol. *7*, 1–16.

Hille, F., Richter, H., Wong, S.P., Bratovič, M., Ressel, S., and Charpentier, E. (2018). The biology of CRISPR-Cas: backward and forward. Cell *172*, 1239–1259.

Joshi, N.A., Fass, J.N. (2011). Sickle: A sliding-window, adaptive, quality-based trimming tool for FastQ files version 1.33.

Koonin, E.V., Makarova, K.S., and Zhang, F. (2017). Diversity, classification and evolution of CRISPR-Cas systems. Curr. Opin. Microbiol. *37*, 67–78.

Koskella, B., and Brockhurst, M.A. (2014). Bacteria-phage coevolution as a driver of ecological and evolutionary processes in microbial communities. FEMS Microbiol. Rev. *38*, 916–931.

Krylov, V.N., Bogush, V.G., Ianenko, A.S., and Kirsanov, N.B. (1980). Pseudomonas aeruginosa bacteriophages with DNA structure similar to the DNA structure of Mu1 phage. II. Evidence for similarity between D3112, B3, and B39 bacteriophages: analysis of DNA splits by restriction endonucleases, isolation of D3112 and B3 recombinant phages. Genetika *16*, 975–984.

Landsberger, M., Gandon, S., Meaden, S., Rollie, C., Chevallereau, A., Chabas, H., Buckling, A., Westra, E.R., and Van Houte, S. (2018). Anti-CRISPR phages cooperate to overcome CRISPR-Cas immunity. Cell *174*, 908–916.e12.

Li, H., and Durbin, R. (2009). Fast and accurate short read alignment with Burrows-Wheeler Transform. Bioinformatics *25*, 1754–1760.

Li, H., Handsaker, B., Wysoker, A., Fennell, T., Ruan, J., Homer, N., Marth, G., Abecasis, G., and Durbin, R.; 1000 Genome Project Data Processing Subgroup (2009). The sequence alignment/map format and SAMtools. Bioinformatics *25*, 2078–2079.

Magoč, T., and Salzberg, S.L. (2011). FLASH: fast length adjustment of short reads to improve genome assemblies. Bioinformatics *27*, 2957–2963.

Mahmoudabadi, G., Milo, R., and Phillips, R. (2017). Energetic cost of building a virus. Proc. Natl. Acad. Sci. USA *114*, E4324–E4333.

Martin, M. (2011). Cutadapt removes adapter sequences from high-throughput sequencing reads. EMBnet J. *17*, 10–12.

Medaney, F., Dimitriu, T., Ellis, R.J., and Raymond, B. (2016). Live to cheat another day: bacterial dormancy facilitates the social exploitation of beta-lactamases. ISME J. *10*, 778–787.

Mendoza, S.D., Nieweglowska, E.S., Govindarajan, S., Leon, L.M., Berry, J.D., Tiwari, A., Chaikeeratisak, V., Pogliano, J., Agard, D.A., and Bondy-Denomy, J. (2019). A bacteriophage nucleus-like compartment shields DNA from CRISPR nucleases. Nature. Published online December 9, 2019.

Morley, D., Broniewski, J.M., Westra, E.R., Buckling, A., and Van Houte, S. (2017). Host diversity limits the evolution of parasite local adaptation. Mol. Ecol. *26*, 1756–1763.

Nakamura, M., Srinivasan, P., Chavez, M., Carter, M.A., Dominguez, A.A., La Russa, M., Lau, M.B., Abbott, T.R., Xu, X., Zhao, D., et al. (2019). Anti-CRISPR-mediated control of gene editing and synthetic circuits in eukaryotic cells. Nat. Commun. *10*, 194.

Nussenzweig, P.M., and Marraffini, L.A. (2018). Viral teamwork pushes CRISPR to the breaking point. Cell *174*, 772–774.

R Core Team (2014). R: A language and environment for statistical computing (R Foundation for Statistical Computing).

Raymond, B., West, S.A., Griffin, A.S., and Bonsall, M.B. (2012). The dynamics of cooperative bacterial virulence in the field. Science *337*, 85–88.

Rognes, T., Flouri, T., Nichols, B., Quince, C., and Mahé, F. (2016). VSEARCH: a versatile open source tool for metagenomics. PeerJ *4*, e2584.

Stanley, S.Y., Borges, A.L., Chen, K.H., Swaney, D.L., Krogan, N.J., Bondy-Denomy, J., and Davidson, A.R. (2019). Anti-CRISPR-associated proteins are crucial repressors of anti-CRISPR transcription. Cell *178*, 1452–1464.e13.

Stanley, S.Y., and Maxwell, K.L. (2018). Phage-encoded anti-CRISPR defenses. Annu. Rev. Genet. *52*, 445–464.

Thavalingam, A., Cheng, Z., Garcia, B., Huang, X., Shah, M., Sun, W., Wang, M., Harrington, L., Hwang, S., Hidalgo-Reyes, Y., et al. (2019). Inhibition of CRISPR-Cas9 ribonucleoprotein complex assembly by anti-CRISPR AcrIIC2. Nat. Commun. *10*, 2806.

Van Houte, S., Ekroth, A.K., Broniewski, J.M., Chabas, H., Ashby, B., Bondy-Denomy, J., Gandon, S., Boots, M., Paterson, S., Buckling, A., and Westra, E.R. (2016). The diversity-generating benefits of a prokaryotic adaptive immune system. Nature *532*, 385–388.

Vorontsova, D., Datsenko, K.A., Medvedeva, S., Bondy-Denomy, J., Savitskaya, E.E., Pougach, K., Logacheva, M., Wiedenheft, B., Davidson, A.R., Severinov, K., and Semenova, E. (2015). Foreign DNA acquisition by the I-F CRISPR-Cas system requires all components of the interference machinery. Nucleic Acids Res. *43*, 10848–10860.

Wang, P.W., Chu, L., and Guttman, D.S. (2004). Complete sequence and evolutionary genomic analysis of the *Pseudomonas aeruginosa* transposable bacteriophage D3112. J. Bacteriol. *186*, 400–410.

Westra, E.R., Van Houte, S., Oyesiku-Blakemore, S., Makin, B., Broniewski, J.M., Best, A., Bondy-Denomy, J., Davidson, A., Boots, M., and Buckling, A. (2015). Parasite exposure drives selective evolution of constitutive versus inducible defense. Curr. Biol. *25*, 1043–1049.

STAR★METHODS

KEY RESOURCES TABLE

REAGENT or RESOURCE	SOURCE	IDENTIFIER
Bacterial and Virus Strains		
P. aeruginosa UCBPP-PA14	Cady et al., 2012	RefSeq: NC_008463.1
P. aeruginosa UCBPP-PA14 *csy3*::*LacZ*	Cady et al., 2012	N/A
P. aeruginosa UCBPP-PA14 BIM-1sp	Westra et al., 2015	N/A
P. aeruginosa UCBPP-PA14 BIM-2sp	Landsberger et al., 2018	N/A
P. aeruginosa UCBPP-PA14 *csy3*::*LacZ* surface mutant Sm	Westra et al., 2015	N/A
P. aeruginosa PAO1::*spycas9*	Mendoza et al., 2019	N/A
P. aeruginosa PAO1::*spycas9* transformed with pJB1-positive control	This study	Table S1
P. aeruginosa PAO1::*spycas9* transformed with pJB1-negative control	This study	Table S1
P. aeruginosa PAO1::*spycas9* transformed with pJB1-anti-AcrIF1	This study	Table S1
P. aeruginosa PAO1::*spycas9* transformed with pJB1-anti-AcrIF4	This study	Table S1
P. aeruginosa PAO1::*spycas9* transformed with pJB1-anti-Acr(-)	This study	Table S1
E. coli DH5α	Thermo Fisher Scientific	Cat#18265017
DMS3*vir*	Cady et al., 2012	N/A
DMS3*vir-acrIF1*	Van Houte et al., 2016	N/A
DMS3*vir-acrIF4*	Landsberger et al., 2018	N/A
DMS3*vir-Δacr*	This study	N/A
D3112	Kryvlov et al., 1980	RefSeq: NC_005178.1
DMS3	O'Toole Lab	RefSeq: NC_008717.1
Deposited Data		
Sequence data	European Nucleotide Archive	ENA: PRJEB29041
Raw data	Mendeley Data	https://doi.org/10.17632/g3ffrjz4dy.1
Oligonucleotides		
Oligonucleotides – spacer cloning	IDT	Table S1
PCR Primers	IDT	Table S2
Recombinant DNA		
pJB1	Mendoza et al., 2019	N/A
pJB1-positive control	This study	Table S1
pJB1-negative control	This study	Table S1
pJB1-anti-AcrIF1	This study	Table S1
pJB1-anti-AcrIF4	This study	Table S1
pJB1-anti-Acr(-)	This study	Table S1
pHERD20T	Davidson lab	GenBank: EU603324.1
Software and Algorithms		
Cutadapt version 1.2.1	Martin, 2011	https://github.com/marcelm/cutadapt
Sickle version 1.200	Joshi and Fass, 2011	https://github.com/najoshi/sickle
Flash version 1.2.11	Magoč and Salzberg 2011	https://ccb.jhu.edu/software/FLASH/
QIIME2 platform (version 2018.2)	Bolyen et al., 2019	https://qiime2.org/
Vsearch	Rognes et al., 2016	https://github.com/torognes/vsearch

(*Continued on next page*)

Continued		
REAGENT or RESOURCE	SOURCE	IDENTIFIER
CRISPRDetect	Biswas et al., 2016	http://crispr.otago.ac.nz/CRISPRDetect/predict_crispr_array.html
Bwa (version 0.7.17)	Li and Durbin, 2009	http://bio-bwa.sourceforge.net/
Samtools (1.3.1)	Li et al., 2009	http://samtools.sourceforge.net/
R (version 3.5.1)	R Core Team, 2014	https://www.R-project.org/
Primer Express 3.0.1	Thermo Fisher Scientific	Cat#4363991
QuantStudio™ Real-Time PCR Software v1.3	Thermo Fisher Scientific Applied Biosystems	https://www.thermofisher.com/uk/en/home/global/forms/life-science/quantstudio-6-7-flex-software.html
Mathematica version 11.2	Wolfram	https://www.wolfram.com/mathematica/quick-revision-history.html
Prism 8.3.0	Graph Pad	https://www.graphpad.com/scientific-software/prism/
Other		
Illumina sequencing	MiSeq platform	https://emea.illumina.com/systems/sequencing-platforms/miseq/specifications.html
QuantStudio™ 7 Flex Real-Time PCR System	Thermo Fisher Scientific Applied Biosystems	Cat#4485690

LEAD CONTACT AND MATERIALS AVAILABILITY

Further information and requests for resources and reagents should be directed to and will be fulfilled by the Lead Contact, Anne Chevallereau (A.Chevallereau@exeter.ac.uk).

All unique/stable reagents generated in this study are available from the Lead Contact without restriction.

EXPERIMENTAL MODEL AND SUBJECT DETAILS

Bacteria

The wild-type strain UCBPP-PA14 of *Pseudomonas aeruginosa* (WT), the derived strains carrying 1 or 2 spacers targeting phage DMS3*vir* (BIM-1sp and BIM-2sp, respectively), the strain UCBPP-PA14 *csy3::lacZ* (CRISPR-KO) which CRISPR-Cas system is not functional and the derived surface mutant (Sm) strain were used throughout this study and are described in Landsberger et al. (2018) and references therein. *Escherichia coli* strain DH5α was used to construct and amplify guide-RNA expression plasmids, which were subsequently transformed into *P. aeruginosa* PAO1*::spycas9* carrying the *cas9* gene of *Streptococcus pyogenes* under the control of an arabinose-inducible promoter (described in Mendoza et al., 2019).

Bacteria were routinely cultured at 37°C either in Lysogeny Broth (LB) or M9 minimal medium (22 mM Na_2HPO_4; 22 mM KH_2PO_4; 8.6 mM NaCl; 20 mM NH_4Cl; 1 mMMgSO$_4$; 0.1 mM $CaCl_2$) supplemented with 0.2% glucose. When appropriate (for plasmids maintenance and expression), LB was supplemented with either 100 $\mu g.ml^{-1}$ ampicillin (*E. coli* DH5α) or 50 $\mu g.ml^{-1}$ gentamicin (PAO1*::spycas9*) and 0.1 % (w/v) arabinose.

Phages

The *Mu*-like virulent phage DMS3*vir* was used throughout this work (described in Cady et al. (2012)). DMS3*vir* infects strains PAO1*::spycas9*, PA14 WT and CRISPR-KO, but not BIM-1sp and BIM-2sp. Phage isogenic variants carrying anti-CRISPR genes, namely DMS3*vir-acrIF1* and DMS3*vir-acrIF4*, were described previously (Landsberger et al., 2018; Van Houte et al., 2016). Phage D3112 (Krylov et al., 1980; Wang et al., 2004), genetically distinct from DMS3*vir* but using the same bacterial receptor (pilus), was used to analyse the evolution of bacterial resistance. Phage stocks were obtained from lysates prepared on PA14 CRISPR-KO and stored at 4°C.

METHOD DETAILS

Individual Phage Infection Assays

Glass vials containing 3 ml of M9 + 0.2% glucose medium were inoculated with approximately 10^7 colony forming units (CFUs) from fresh overnight cultures of WT or CRISPR-KO strains and infected with either phage DMS3*vir*, DMS3*vir-acrIF1* or DMS3*vir-acrIF4* at an initial multiplicity of infection (MOI) of ~0.01. Infected cultures were then incubated at 37°C under agitation and transferred daily (1:100 dilution) into fresh medium. Each experiment was performed in 6 replicates. Phage and bacterial concentrations were assessed every day for 3 days by spot assays and cell plating, respectively.

Analysis of Evolved Phage Resistance

High-Throughput Sequencing of CRISPR Arrays

Total DNA was extracted from samples of WT bacterial cultures individually infected with either DMS3*vir*, DMS3*vir-acrIF1* or DMS3*vir-acrIF4*, at 3 days post infection (dpi), using QIAamp DNA Mini kit (Qiagen), according to manufacturer's instructions. Quality and concentration of DNA samples were verified on a 0.5% agarose gel and assessed by Qubit. To generate amplicons for sequencing, the following primers were used:

5'-GGCGCTGGAGCCCTTGGGGCTTGG and 5'-GCGGCTGCCGGTGGTAGCGGGTG for CRISPR array 1;

5′-GCTCGACTACTACAACGTCCGGC and 5′-GGGTTTCTGGCGGGAAAAACTCGG for CRISPR array 2.

Libraries were prepared by the Centre for Genomic Research (University of Liverpool, UK) and 2×250 bp paired-end reads generated on an Illumina MiSeq platform.

Sequenced reads were trimmed for the presence of Illumina adapter sequences using Cutadapt version 1.2.1 (Martin, 2011). The option -O 3 was used, so the 3′ end of any reads which match the adapter sequence for 3 bp or more are trimmed. Reads were further trimmed using Sickle version 1.2 (https://github.com/najoshi/sickle) with a minimum window quality score of 20. Reads shorter than 20 bp after trimming were removed. Reads were merged with Flash version 1.2.11 (Magoč and Salzberg 2011) and a further 5 bases were trimmed from the 5′ end of each read, following additional quality checks. The resulting read length distributions were determined directly with an Awk expression. Merged reads were then processed using the Qiime2 platform (version 2018.2). Additional quality filtering was done using the default settings based on sequence quality scores (minimum phred score = 4, maximum number of consecutive low scores = 3, minimum length of sequence after filtering = 75% of the original read). Sequences were dereplicated and clustered at 99% similarity using Vsearch (Rognes et al., 2016). Spacers from the clustered reads were predicted using a modified version of CRISPRDetect (Biswas et al., 2016) and extracted using a Perl script. Spacers were mapped to the DMS3*vir* genome (based on NCBI RefSeq: NC_008717 edited to match the sequence described in Cady et al., 2012) using bwa (version 0.7.17) and samtools (1.3.1). The resulting BAM files were plotted in R (version 3.5.1).

Phenotypic Analyses

To determine which phage-resistance mechanisms evolved in WT bacterial populations, either upon individual or mixed-infections, 24 individual colonies (per replicate) were picked and grown in 200 μl of LB broth for 3h at 37°C and 5 μl of these cultures were spotted on LB agar plates, in triplicate. Two microliters (approximately 10^5 plaque forming units (PFUs)) of either the ancestral phage (i.e. DMS3*vir*, DMS3*vir-acrIF1* or DMS3*vir-acrIF4*), an alternative phage D3112 or LB broth (negative control) were dropped on top of bacterial spots. These phenotypic assays allowed to determine whether a given clone was (i) phage-sensitive (lysed by both the ancestral and alternative phages), (ii) resistant through surface modification (resistant to both ancestral and alternative phages) or (iii) resistant through CRISPR-immunity (resistant to ancestral phage, sensitive to alternative phage). For the latter, CRISPR-resistance was further confirmed by testing clones for acquisition of spacers by PCR using primers 5′-CTAAGCCTTGTACGAAGTCTC and 5′-CGCCGAAGGCCAGCGCGCCGGTG (for CRISPR array 1) and 5′-GCCGTCCAGAAGTCACCACCCG and 5′-TCAGCAAGTTAC GAGACCTCG (for CRISPR array 2).

Bacterial Competition Experiments

Glass vials with 6 ml M9 + 0.2% glucose were inoculated with approximately 2.10^7 CFUs from a 1:1 mixture of overnight cultures (grown in M9 medium + 0.2% glucose) either of phage-resistant strains BIM-2sp and Sm, or of phage-sensitive strains WT and CRISPR-KO. Phages (DMS3*vir*, DMS3*vir-acrIF1* or DMS3*vir-acrIF4*) were then added to each glass vial at a MOI of 0.01 (for the WT x CRISPR-KO competition) or 25 (for the BIM-2sp x Sm competition). Control competition experiments in the absence of phages were performed in parallel. All competition experiments were performed in 6 replicates. Mixed-cultures were transferred daily (1:100 dilution) into fresh medium. At 0, 1 and 3 days after the start of the experiment, samples were taken and cells were serially diluted in M9 medium and plated on LB agar supplemented with 50 $mg.ml^{-1}$ X-gal (to allow discrimination between WT or BIM-2sp (white) and CRISPR-KO or Sm (blue) strains). Phage concentrations were also monitored at 0, 1 and 3 days using spot assays. Relative frequencies (fractions) of competing strains were determined through colony numbers and used to calculate the relative fitness according to formula below:

$$Relative\ fitness\ WT_{t=x} = \frac{(Fraction\ WT_{t=x}) \times (1 - Fraction\ WT_{t=0})}{(Fraction\ WT_{t=0}) \times (1 - Fraction\ WT_{t=x})}$$

Phage Competition Experiments

Mixed Phage Infections

Phage mixtures (1:1) of either DMS3*vir-acrIF1*:DMS3*vir*, DMS3*vir*:DMS3*vir-acrIF4* or DMS3*vir-acrIF1*:DMS3*vir-acrIF4* were used to infect fresh cultures of either CRISPR-KO, WT, BIM-1sp or BIM-2sp (approx. 4.10^6 $CFU.ml^{-1}$ in 6 mL of M9 + 0.2% glucose medium, verified by cell plating), each in 6 replicates. Mixed-infections were carried out at a MOI of ~0.02 on CRISPR-KO and WT strains, at MOIs of ~0.01, 0.1 and 1 on BIM-1sp and at MOIs of ~1 and 50 on BIM-2sp. Cultures were transferred daily (1:100 dilution) into fresh medium and samples were taken at 0, 1, 2 and 3 dpi to monitor the concentrations of each phage population. Upon chloroform

extraction (addition of 1:10 v/v chloroform to cultures, vortex for 30 s and pellet cell debris and chloroform at 4°C), total phage samples were serially diluted in M9 medium and spot assays were performed on PAO1::*spycas9* indicator strains (see below for description). This allowed to distinguish competing phages and hence to determine the concentrations of each type of phage genotype in the population.

Construction of PAO1::spycas9 Indicator Strains

The pJB1 plasmid (described in Mendoza et al., 2019) harbours a BsaI site for insertion of a desired spacer sequence, a crRNA repeat sequence and a tracrRNA sequence. Upon digestion with BsaI, annealed and phosphorylated oligonucleotides containing the spacer sequence of interest flanked by BsaI sites were ligated into pJB1. Strain PAO1*::spycas9* was transformed with pJB1 expression vectors by electroporation. Briefly, an indicator strain PAO1*::spycas9* carrying a pJB1 expression vector produces a crRNA::tracrRNA-loaded SpyCas9 protein that targets a protospacer complementary to the spacer cloned into the pJB1 vector. As a result, a phage carrying this protospacer cannot produce plaques on that indicator strain. All indicator strains (and corresponding spacer sequences) are listed in Table S1.

Determination of Relative Fitness of Competing Phages by qPCR

Relative frequencies of each phage in co-cultures were measured at 0, 1, 2 and 3 dpi with qPCR (using specific primer sets) allowing the calculation of phages' relative fitness. Primer pairs were designed using Primer Express 3.0.1 and are listed in Table S2. Each primer pair was tested against the 2 other phages and non-specific amplification could not be detected. Total phage samples obtained upon chloroform extractions were used as templates. Each qPCR reaction was prepared following manufacturer's recommendations and composed of 7.5 μl Brilliant III Ultra-Fast SYBR® Green QPCR Master Mix (Agilent Technologies, Santa Clara, CA, United States of America), 0.3 μl of provided reference dye (freshly diluted 1:50 in PCR-grade water, final concentration 300 nM), 1.5-μl primer pair (4.5μM each), 0.15-μl bovine serum albumin (20 mg.ml^{-1}), 3.75 μl undiluted phage sample (or standard phage solutions or water), and PCR-grade water to a total volume of 15 μl. For each primer set, standard reactions (six ten-fold dilutions of pure matching-phage stock solutions (10^3 to 10^8 PFU.ml^{-1}) in PCR-grade water) and negative controls (either water or 10^8 PFUs of non-matching phages) were systematically included and performed in triplicate. Two qPCR reactions were performed on each sample (each reaction being specific to one or the other phage in the sample). All samples were run at the same time, on a 384-well plate, to avoid between-run variations and to ensure that all samples were analysed against the same standards curves. The qPCR program was 95°C for 10 min, followed by 40 cycles of 95°C for 15 s and 60°C for 20 s and was run on QuantStudio™ 7 Flex Real-Time PCR System (Applied Biosystems™). Provided that the efficiencies of the reactions were between 90% and 110%, the threshold cycle (C_T) was used to calculate the quantity of the targeted phage in each sample (deduced from standard curves, computed by QuantStudio™ Real-Time PCR Software v1.3). Average quantities (Q) - for the 6 biological replicates of each competition – were then used to calculate phage relative frequencies (fraction, see below), allowing to further calculate phage relative fitness (same equation as described in the bacterial competition section above).

$$Fraction\ phage\ 1 = \frac{Q_{phage1}}{Q_{phage1} + Q_{phage2}}$$

Fitness Costs Associated with *acr* Operon

Potential fitness costs associated with *acr* operon were assessed by competing a phage deletion mutant lacking the entire *acr* operon (i.e. promoter, Acr coding sequence and *aca1* gene) with an isogenic phage carrying the operon (with AcrIE3 or AcrIF1 or AcrIF4 coding sequence) in absence of selection (i.e. on CRISPR-KO host). Phage competition experiments were performed as indicated above.

Construction of Phage Deletion Mutants

Recombination cassettes containing in-frame deletions of the *acr* operon bordered by ~ 500–650 bp flanking regions at each side were generated and inserted into the shuttle vector pHERD20T using a Gibson assembly protocol (as described in Borges et al., 2018). The 'up' and 'down' fragments of the cassette were amplified from phage DMS3*vir-acrIE3* using the following primers (respectively): 5′-TACCCATGGGATCTGATAAGAATTCGAGCTATCCGTCTGCGCGGCGAGATA (forward), 5′- CGTGTAGCGCGTTTGCGG GCGGATCAGGTGAAGGCACAGTGTGCCGCTTGTC (reverse) and 5′-TCACCTGATCCGCCCGCAAAC (forward), 5′-GACGGCCA GTGCCAAGCTTGCATGCCTGCACATTCGAAATCGAGGAAGCGGC (reverse). The PA14 CRIPSR-KO strain was transformed with this recombination vector by electroporation and transformants were infected with DMS3 to generate recombinant temperate phages (as described in Borges et al., 2018). The resulting DMS3 deletion mutants were essayed for their inability to interfere with CRISPR-Cas targeting, and the genomic region normally enclosing the *acr* operon was PCR amplified and sequenced to confirm correct recombination. CRISPR-KO lysogens of DMS3 deletion mutants were used to truncate gene DMS3-1 (c-repressor) as described in Cady et al. (2012), yielding recombinant lytic phages carrying deletion of *acr* operon (referred to as Δ*acr*).

Efficiency of Centre of Infection (ECOI) Assays

Overnight cultures of CRISPR-KO and BIM-1sp were OD-adjusted and 20 μl were used to inoculate 180 μl of fresh M9 + 0.2% glucose in a 96-well plate. After 2h-growth at 37°C, 20-μl aliquots were sampled to measure uninfected bacterial concentration and phages (DMS3*vir*, DMS3*vir-acrIF1* or DMS3*vir-acrIF4*) were added at MOI of ~1 and incubated for 30 min (adsorption). Cultures were then washed three times with M9 salts to remove free phages, serial diluted and 5 μl were spotted onto LB agar (concentration of

pre-adsorbed bacteria) or onto a lawn of CRISPR-KO strain. Each plaque formed on the CRISPR-KO lawn indicates a successful infection of a single pre-adsorbed cell (i.e. a centre of infection). ECOI was calculated as follows:

$$EOIC = \frac{[Centres\ of\ Infection]}{[Pre-adsorbed\ cells]xMOI}$$

By normalizing the number of successful infections on a CRISPR-resistant host by that on CRISPR-KO host, this assay provides a direct measure of the success rate of the first infection of a CRISPR-resistant host by a phage, which reflects the value of φ.

$$\varphi \approx \frac{ECOI_{BIM-1sp}}{ECOI_{CRISPR-KO}}$$

Mathematical Modelling

We extended our previously described epidemiological model (Landsberger et al., 2018), to model the population dynamics of Acr-phages in an initially sensitive host population that can evolve CRISPR resistance. All simulations were performed in the software Mathematica version 11.2.

Bacteria may either be sensitive (the density of these bacteria is denoted $W(t)$), or may have evolved CRISPR-resistance after the incorporation of a spacer targeting the phage (the density of these bacteria is noted $R(t)$), or may be in an immunosuppressed state (the density of these bacteria is noted $S(t)$).

Initially the host population is homogeneous, consisting exclusively of sensitive bacteria. Bacteria grow at a maximal rate r, but this growth is limited by the density of bacteria (where k measures the intensity of density dependence), and bacteria die with mortality rate m. At $t = 0$, an inoculum of free Acr-positive phages with density $V_1(0)$ is introduced in the host population, or an inoculum of Acr-negative phages with density $V_2(0)$, or an equal mix of Acr-positive and Acr-negative phages, such that $V_1(0) = V_2(0)$.

All free phage particles adsorb to the bacteria at a rate a. When a free phage adsorbs to a sensitive bacterium, two outcomes are possible:

(i) with probability $1 - A$, the phage lyses the host, leading to the release of B new phage particles
(ii) with probability A, the bacterium acquires CRISPR-based resistance, leading to the destruction of the phage genome

When an Acr-negative phage particle adsorbs to a CRISPR-resistant bacterium, its genome is destroyed. When an Acr-positive phage adsorbs to a CRISPR-resistant bacterium, three outcomes are possible, as described in our previous model (Landsberger et al., 2018):

(i) with probability ρ, the Acr-positive phage genome is destroyed prior to expression of the *acr* gene, with no change in bacterial resistance (i.e. no immunosuppression). Hence, ρ is a measure of bacterial resistance and increases with the number of spacers targeting the phage. We assume ρ is governed by the host and independent of the Acr.
(ii) with probability $(1 - \rho)\varphi$,the Acr-positive phage lyses the cell, with the release of B new Acr-positive phage particles. Hence, the greater φ, the greater is the ability of the Acr-positive phage to bypass the CRISPR-Cas immune system.
(iii) with probability $(1 - \rho)(1 - \varphi)$, the Acr-positive phage fails to complete its lytic cycle but produces some Acr proteins before its genome is cleaved, which block bacterial CRISPR-resistance and cause the bacterium to become immunosuppressed. This state is reversible and immunosuppressed bacterium become resistant again at rate γ. Hence, the smaller γ, the longer the bacterium remains in the immunosuppressed state.

If an immunosuppressed bacterium is infected by a phage, the absence of resistance allows the phage to complete its lytic cycle, even if it does not encode an Acr. This yields the following set of ordinary differential equations (see Figure 5A):

$$\dot{W}(t) = rW(t)(1-kN(t)) - (aV_1(t) + aV_2(t) + m)W(t)$$

$$\dot{R}(t) = AaV(t)W(t) + rR(t)(1-kN(t)) - (a\,(1-\rho)V_1(t) + m)R(t) + \gamma S(t)$$

$$\dot{S}(t) = a(1-\rho)(1-\varphi)V_1(t)R(t) - (a\,V(t) + m + \gamma)S(t)$$

$$\dot{V}_1(t) = a(1-\rho)\varphi B\,V_1(t)R(t) + aB\,V_1(t)(S(t) + (1-A)W(t)) - a\,N(t)V_1(t)$$

$$\dot{V}_2(t) = aB\,V_2(t)(S(t) + (1-A)W(t)) - a\,N(t)V_2(t)$$

With $N(t) = W(t) + R(t) + S(t)$, and $V(t) = V_1(t) + V_2(t)$.

We used the above model to monitor the infection dynamics following infection with an equal mix of Acr-positive phages (V_1) and Acr-negative phages (V_2) that cannot infect resistant bacteria, with different parameter values φ and γ, reflecting different strength of Acr. All the parameter values are given in the legends of related figures.

QUANTIFICATION AND STATISTICAL ANALYSIS

All graphs of experimental data and statistical analyses were generated with GraphPad Prism 7. Statistical details of experiments are indicated in the figure legends. After verifying that the data (log-transformed when appropriate) were not inconsistent with a Gaussian distribution (Shapiro-Wilk normality test), one-tailed t-tests were used to determine whether experimental values (i.e. relative fitness or phage amplification) significantly differed from a theoretical value (e.g. 1), and two-tailed unpaired t-tests were used to compare groups with each other, where appropriate. Each group was composed of 6 values (i.e. 6 independent biological replicates) and 95% confidence levels were used in all statistical tests. In all cases, observed differences were considered significant when p-values were less than a Bonferroni-corrected threshold of $0.05/a$, where a is the number of comparisons.

DATA AND CODE AVAILABILITY

The accession number for the amplicon sequencing data reported in this paper is ENA: PRJEB29041. Source data for Figures 1, 2, 3, 4, 6, S1, S2, and S4 are available at Mendeley Data (https://doi.org/10.17632/g3ffrjz4dy.1).

Cell Host & Microbe, Volume *27*

Supplemental Information

Exploitation of the Cooperative Behaviors of Anti-CRISPR Phages

Anne Chevallereau, Sean Meaden, Olivier Fradet, Mariann Landsberger, Alice Maestri, Ambarish Biswas, Sylvain Gandon, Stineke van Houte, and Edze R. Westra

Supplementary Figures

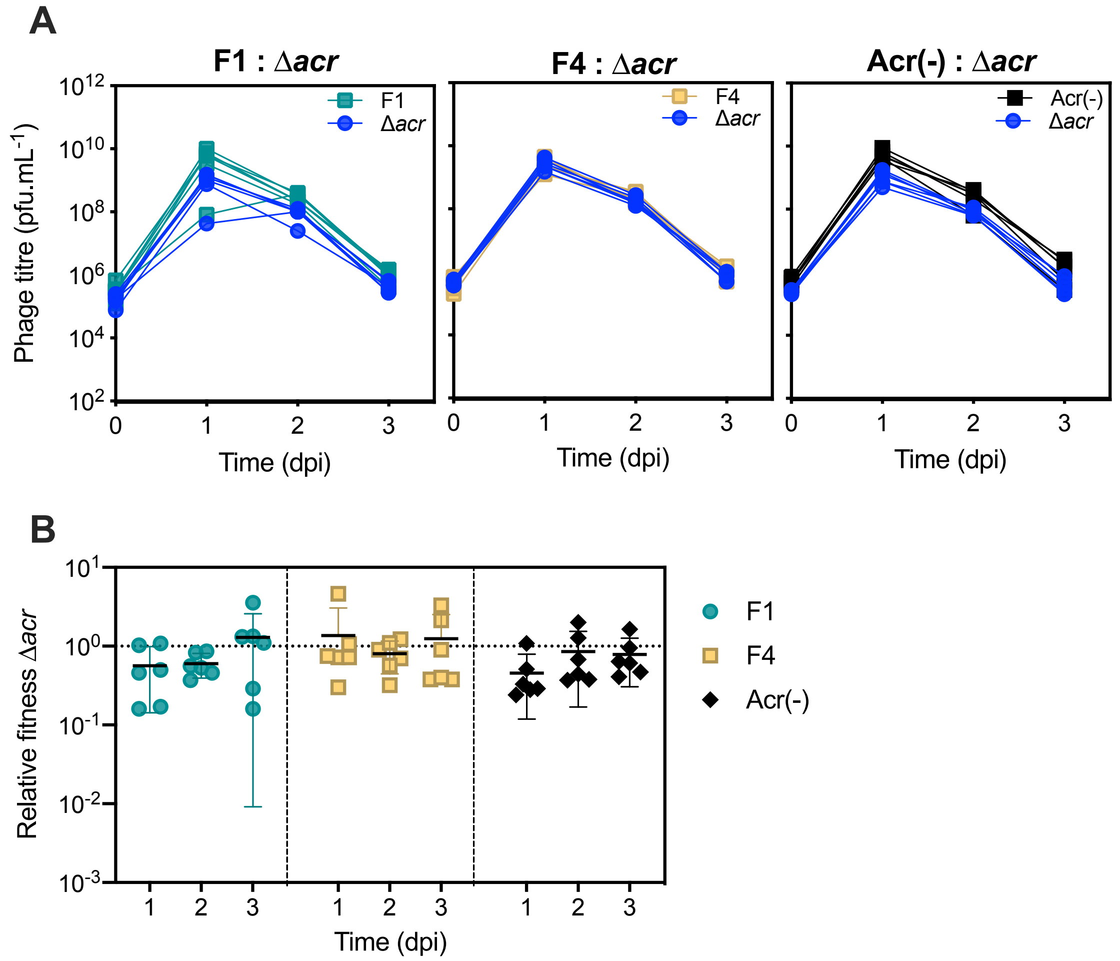

Figure S1. Carrying *acr* operon is not associated with fitness costs. Related to Figure 2. Phage mutants lacking the *acr* operon (Δ*acr*) were co-cultured for 3 days with either Acr-positive phages encoding the strong AcrIF1 (F1) or the weak AcrIF4 (F4) or with Acr-negative phages encoding AcrIE3 (Acr(-)), inactive against the CRISPR-Cas system of WT PA14. The host population carries a non-functional CRISPR-Cas system (CRISPR-KO). (**A**) Titres of each phage type were determined by spot assay on indicator strains. **(B)** Fitness values of Δ*acr*-phage relative to that of indicated phage competitors were calculated each day (with fitness at T=0 set as a reference). Panels show individual data (A) and mean (B) from 6 biological replicates. Errors bars show 95% c.i.

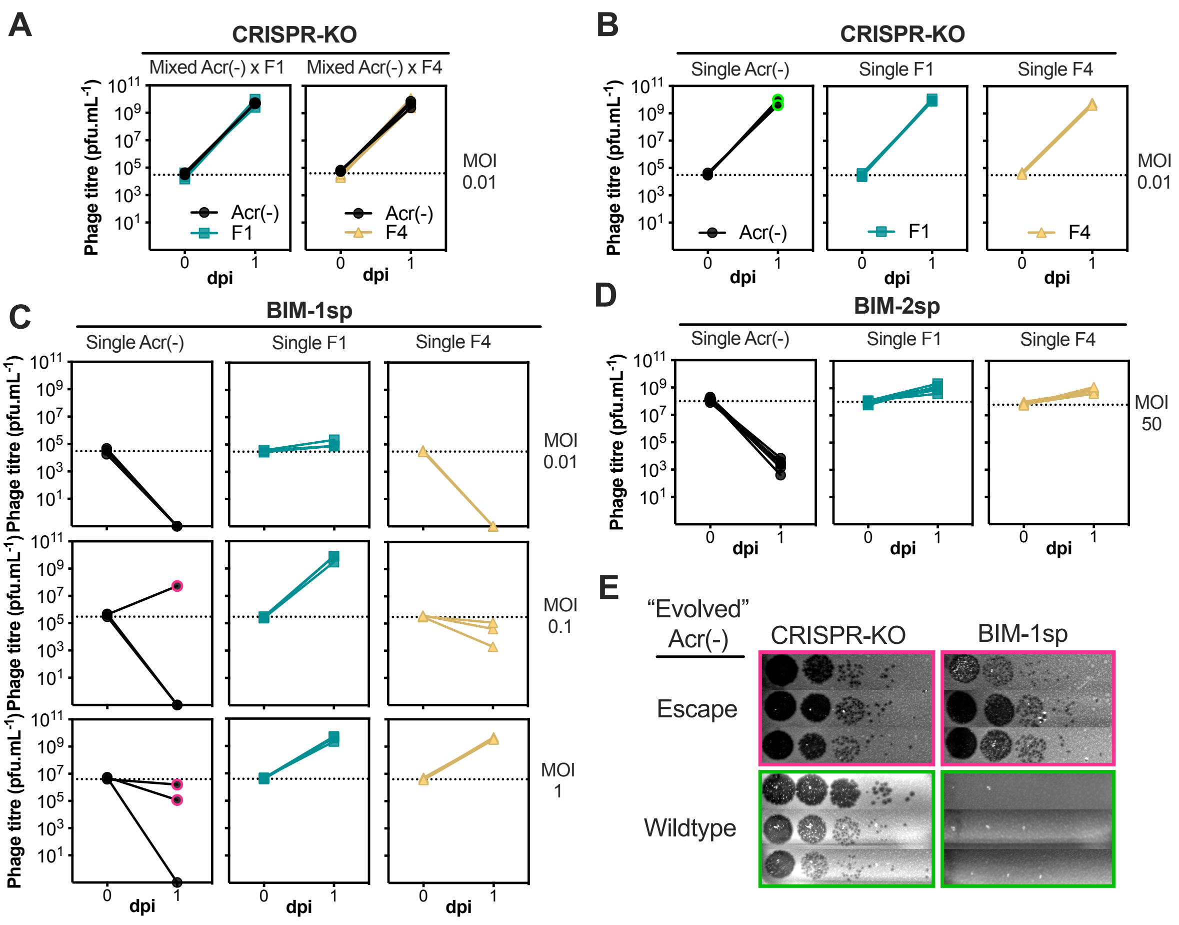

Figure S2. Control phage amplification during individual or mixed infection of CRISPR-KO or BIM host. Related to Figure 4.

Amplification of Acr-negative and Acr-positive phages during (**A**) mixed or (**B**) individual infection of CRISPR-KO host, (**C**) individual infection of CRISPR-resistant host BIM-1sp or (**D**) BIM 2sp. Initial MOI are indicated. (**E**) Acr-negative phages that amplified/maintained after 24h individual infection of CRISPR-KO (green circled dots) or BIM-1sp (pink circled dots) were isolated and the ability of these 'evolved' Acr-negative phages to infect BIM-1sp on their own was assessed (EOP assay). Individual data from 3 biological replicates are shown.

A

Acr(-):F1 – BIM-1sp (MOI 1)

Protospacer 1

ancestral Acr(-)	CC**AACGGCCGACGCTTCTGGGTCGTCGTGAAAGT**
1dpi-Acr(-)_A1	CCAACGGCCGACGCTTCTGGGTCGTCGTGAAAGT
1dpi-Acr(-)_A2	CCAACGGCCGACGCTTCTGGGTCGTCGTGAAAGT
1dpi-Acr(-)_A3	CCAACGGCCGACGCTTCTGGGTCGTCGTGAAAGT
1dpi-Acr(-)_A4	CCAACGGCCGACGCTTCTGGGTCGTCGTGAAAGT
1dpi-Acr(-)_A5	CCAACGGCCGACGCTTCTGGGTCGTCGTGAAAGT
1dpi-Acr(-)_A6	CCAACGGCCGACGCTTCTGGGTCGTCGTGAAAGT

B

Acr(-):F4 – BIM-1sp (MOI 0.1 – MOI 1)

Protospacer 1

ancestral Acr(-)	CC**AACGGCCGACGCTTCTGGGTCGTCGTGAAAGT**
1dpi-Acr(-)_C1	CCAGCGGCCGACGCTTCTGGGTCGTCGTGAAAGT
1dpi-Acr(-)_C2	CGAACGGCCGACGCTTCTGGGTCGTCGTGAAAGT
1dpi-Acr(-)_C3	CCAGCGGCCGACGCTTCTGGGTCGTCGTGAAAGT
1dpi-Acr(-)_C4	CTAACGGCCGACGCTTCTGGGTCGTCGTGAAAGT
1dpi-Acr(-)_C5	CCAACGCCCGACGCTTCTGGGTCGTCGTGAAAGT
1dpi-Acr(-)_C6	CTAACGGCCGACGCTTCTGGGTCGTCGTGAAAGT

C

Acr(-):F1 – BIM-2sp (MOI 1)

Protospacer 1

ancestral Acr(-)	CC**AACGGCCGACGCTTCTGGGTCGTCGTGAAAGT**
1dpi-Acr(-)_B1	CCAACGGCCGACGCTTCTGGGTCGTCGTGAAAGT
1dpi-Acr(-)_B2	CCAACGGCCGACGCTTCTGGGTCGTCGTGAAAGT
1dpi-Acr(-)_B3	CCAACGGCCGACGCTTCTGGGTCGTCGTGAAAGT
1dpi-Acr(-)_B4	CCAACGGCCGACGCTTCTGGGTCGTCGTGAAAGT
1dpi-Acr(-)_B5	CCAACGGCCGACGCTTCTGGGTCGTCGTGAAAGT

Protospacer 2

ancestral Acr(-)	CC**TGGACAACGGCAGCGCGATTCCCCGCGTGACC**
1dpi-Acr(-)_B1	CCTGGACAACGGCAGCGCGATTCCCCGCGTGACC
1dpi-Acr(-)_B2	CCTGGACAACGGCAGCGCGATTCCCCGCGTGACC
1dpi-Acr(-)_B3	CCTGGACAACGGCAGCGCGATTCCCCGCGTGACC
1dpi-Acr(-)_B4	CCTGGACAACGGCAGCGCGATTCCCCGCGTGACC
1dpi-Acr(-)_B5	CCTGGACAACGGCAGCGCGATTCCCCGCGTGACC

Figure S3. Protospacer sequences of Acr-negative phages. Related to Figure 4.

Acr-negative phages that amplified on CRISPR-resistant host carrying (**A,B**) 1 spacer (BIM-1sp) or (**C**) 2 spacers (BIM-2sp) were recovered at 1 dpi and phage protospacers were sequenced. Panels (**A**) and (**C**) show Acr-negative phages grown in presence of strong Acr-phage F1 (isolated from experiments of Fig. 4a and b, respectively (green circled dots)). Panel (**B**) shows Acr-negative phages cultured in presence of weak Acr-phage F4 (isolated from experiment presented in Fig. 4c (pink circled dots)). Protospacers of wildtype DMS3*vir* are indicated in bold letters and protospacer adjacent motifs are underlined. Point escape mutations are highlighted in red in panel b. Sequences shown correspond to phages isolated from 5 or 6 biological replicate experiments.

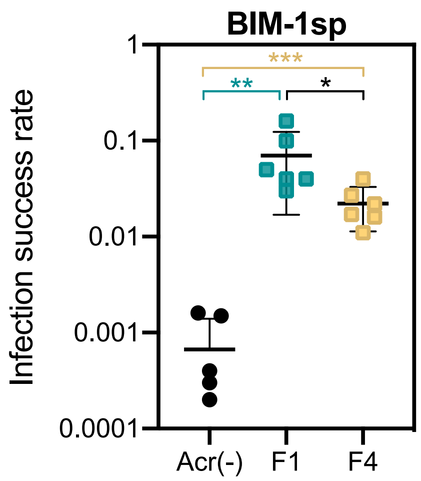

Figure S4. Experimental estimation of parameter ϕ. Related to Figure 5.

An estimation of parameter ϕ was achieved by measuring the ECOI of phages DMS3*vir*, DMS3*vir-acrIF1* and DMS3*vir-acrIF4* (indicated as Acr(-), F1 and F4, respectively) on CRISPR-resistant host BIM-1sp (one targeting spacer) and CRISPR-KO host. Asterisks indicate significant differences (two tailed t-tests): Acr(-)/F1: $p=0.007$, $t_{10}=3.36$; Acr(-)/F4: $p=0.0005$, $t_{10}=5.10$; F1/F4: $p=0.05$, $t_{10}=2.27$. Individual values and means from 6 biological replicates are shown. Error bars represent 95% c.i.

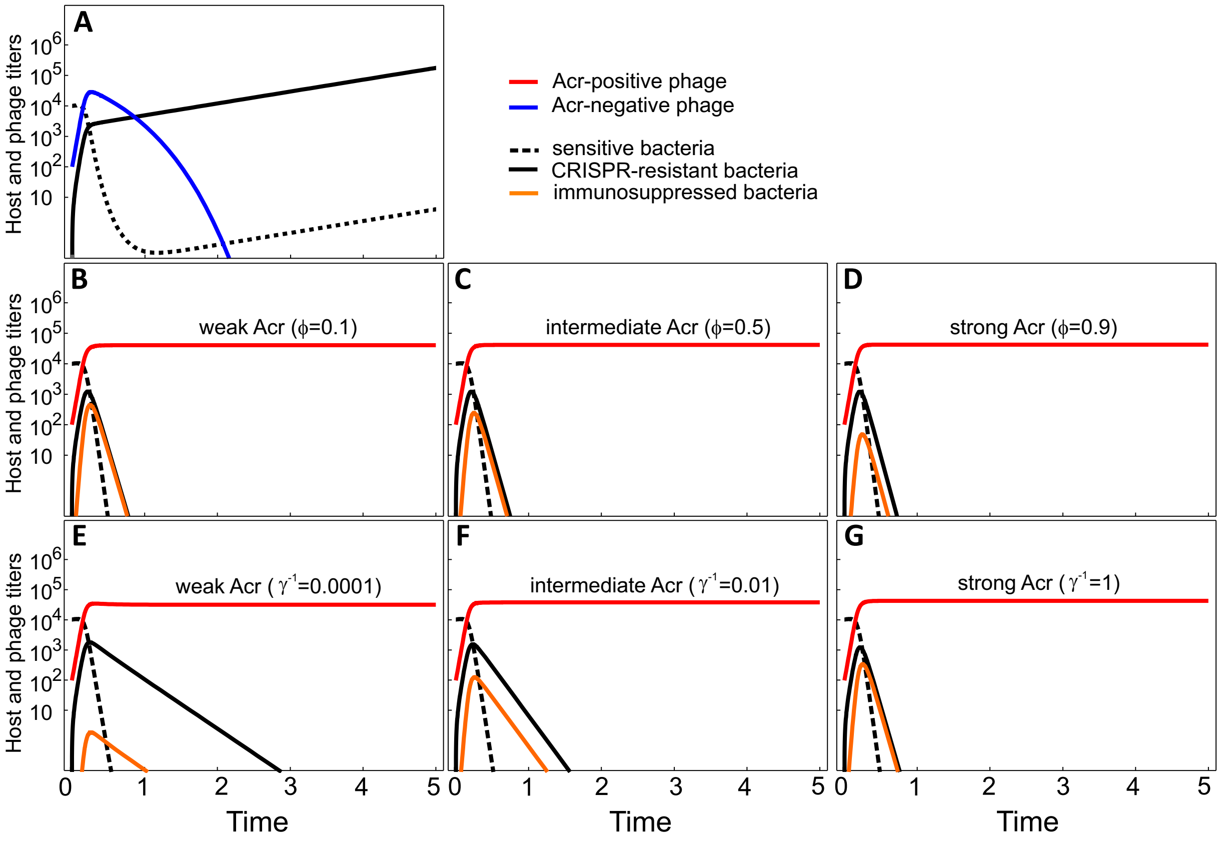

Figure S5. Simulations of phage/host evolutionary and population dynamics during individual infections. Related to Figure 5.

(**A**) Infection dynamics and evolution of CRISPR-resistance (solid black line) upon infection of initially sensitive hosts (dashed line) with 100 Acr-negative phages (blue line), or (**B-G**) upon infection with 100 Acr-positive phages (red lines), with different parameter values for ϕ and γ as follows: (B) $\phi = 0.1$ and $\gamma^{-1} = 0.1$, (C) $\phi = 0.5$ and $\gamma^{-1} = 0.1$, (D) $\phi = 0.9$ and $\gamma^{-1} = 0.1$, (E) $\phi = 0.3$ and $\gamma^{-1} = 0.0001$, (F) $\phi = 0.3$ and $\gamma^{-1} = 0.01$, (G) $\phi = 0.3$ and $\gamma^{-1} = 1$.

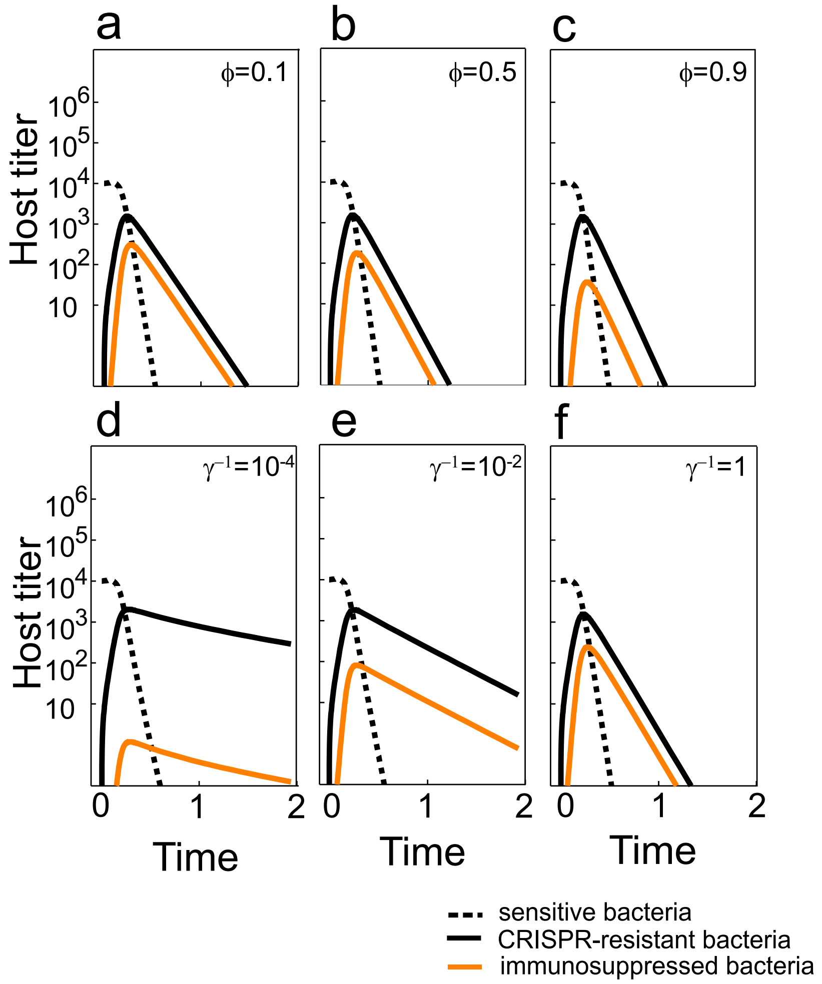

Figure S6. Simulations of host evolutionary and population dynamics during infections with mixed phage populations. Related to Figure 5.

Infection dynamics and evolution of CRISPR-resistance (solid black line) upon infection of initially sensitive hosts (dashed line) with an equal mix of Acr-negative and Acr-positive phage with different parameter values for ϕ and γ as follows: (a) $\phi = 0.1$ and $\gamma^{-1} = 0.1$, (b) $\phi = 0.5$ and $\gamma^{-1} = 0.1$, (c) $\phi = 0.9$ and $\gamma^{-1} = 0.1$, (d) $\phi = 0.3$ and $\gamma^{-1} = 0.0001$, (e) $\phi = 0.3$ and $\gamma^{-1} = 0.01$, (f) $\phi = 0.3$ and $\gamma^{-1} = 1$. Other parameter values: a = 0.001, A = 0.2, B = 5, $\rho = 0.5$.

Table S1. List of PAO1::*spycas9* indicator strains used to monitor individual concentrations of competing phages. Related to STAR Methods.

Indicator strain name (PAO1::*spycas9* + pJB1)	Phage sensitivity	Spacer sequence inserted into pJB1
Positive control	Resistant to all phages	5'-GTCTGCGCGGCGAGATATACG
Anti-AcrIF1	Resistant to DMS3*vir-acrIF1* only	5'-GTCGTAAACGGCAAAAGCGTT
Anti-AcrIF4	Resistant to DMS3*vir-acrIF4* only	5'-GTAAGCAACGGGTGGCAGTGG
Anti-Acr(-)	Resistant to DMS3*vir* only	5'-ATTTGGTGGCGACGCCGGCCG
Negative control	Sensitive to all phages	None

Table S2. List of primers used in this study. Related to STAR Methods.

Primer name	Sequence (5' – 3')
CRISPR array 1_SeqF	GGCGCTGGAGCCCTTGGGGCTTGG
CRISPR array 1_SeqR	GCGGCTGCCGGTGGTAGCGGGTG
CRISPR array 2_SeqF	GCTCGACTACTACAACGTCCGGC
CRISPR array 2_SeqR	GGGTTTCTGGCGGGAAAAACTCGG
CRISPR array 1_F	CTAAGCCTTGTACGAAGTCTC
CRISPR array 1_R	CGCCGAAGGCCAGCGCGCCGGTG
CRISPR array 2_F	GCCGTCCAGAAGTCACCACCCG
CRISPR array 2_R	TCAGCAAGTTACGAGACCTCG
DMS3*vir*_F	ACCTGAGCGAGGATCAATGG
DMS3*vir*_R	CGCGGCGAACCTTCTG
DMS3*vir-acrIF1*_F	CGAAAATGGCAGCAAAATCAA
DMS3*vir-acrIF1*_R	CCAACGCTTTTGCCGTTT
DMS3*vir-acrIF4*_F	GTGGCGCCCTCCATCAT
DMS3*vir-acrIF4*_R	GCAAGCCGAAGTAACCATTCTC
Δ*acr*-Up_F	TACCCATGGGATCTGATAAGAATTCGAGCTATCCGTCTGCGCGGCGAGATA
Δ*acr*-Up_R	CGTGTAGCGCGTTTGCGGGCGGATCAGGTGAAGGCACAGTGTGCCGCTTGTC
Δ*acr*-Down_F	TCACCTGATCCGCCCGCAAAC
Δ*acr*-Down_R	GACGGCCAGTGCCAAGCTTGCATGCCTGCACATTCGAAATCGAGGAAGCGGC
